# A Scheme to Optimize Flow Routing and Polling Switch Selection of Software Defined Networks

**DOI:** 10.1371/journal.pone.0145437

**Published:** 2015-12-21

**Authors:** Huan Chen, Lemin Li, Jing Ren, Yang Wang, Yangming Zhao, Xiong Wang, Sheng Wang, Shizhong Xu

**Affiliations:** Key Laboratory of Optical Fiber Sensing and Communication (Education Ministry of China), University of Electronic Science and Technology of China, Chengdu, Sichuan, China; Nankai University, CHINA

## Abstract

This paper aims at minimizing the communication cost for collecting flow information in Software Defined Networks (SDN). Since flow-based information collecting method requires too much communication cost, and switch-based method proposed recently cannot benefit from controlling flow routing, jointly optimize flow routing and polling switch selection is proposed to reduce the communication cost. To this end, joint optimization problem is formulated as an Integer Linear Programming (ILP) model firstly. Since the ILP model is intractable in large size network, we also design an optimal algorithm for the multi-rooted tree topology and an efficient heuristic algorithm for general topology. According to extensive simulations, it is found that our method can save up to 55.76% communication cost compared with the state-of-the-art switch-based scheme.

## Introduction

To improve the network performance, network operators need to conduct many measurement tasks. Traditionally, there are two types of measurement methods including active measurement and passive measurement. In active measurement, network operators inject probe traffics into the network. This method is easy to deploy but it may affect the performance of the network since extra traffics are introduced. In passive measurement, network operators monitor the performance of network devices such as routers and switches to measure the performance of entire network. Therefore, it has little impact to the network performance. However, the security concerns cannot be ignored since passive measurement requires full access to those network devices.

The emergence of Software Defined Network (SDN) provides more opportunities for network measurement. It provides Application Programming Interfaces (API) to query the network performance statistics of each flow or each switch. Accordingly, it greatly reduces the amount of probing traffic in conventional active measurement techniques, and avoids fully accessing the network components as in previous passive measurement methods.

**Fig 1:**
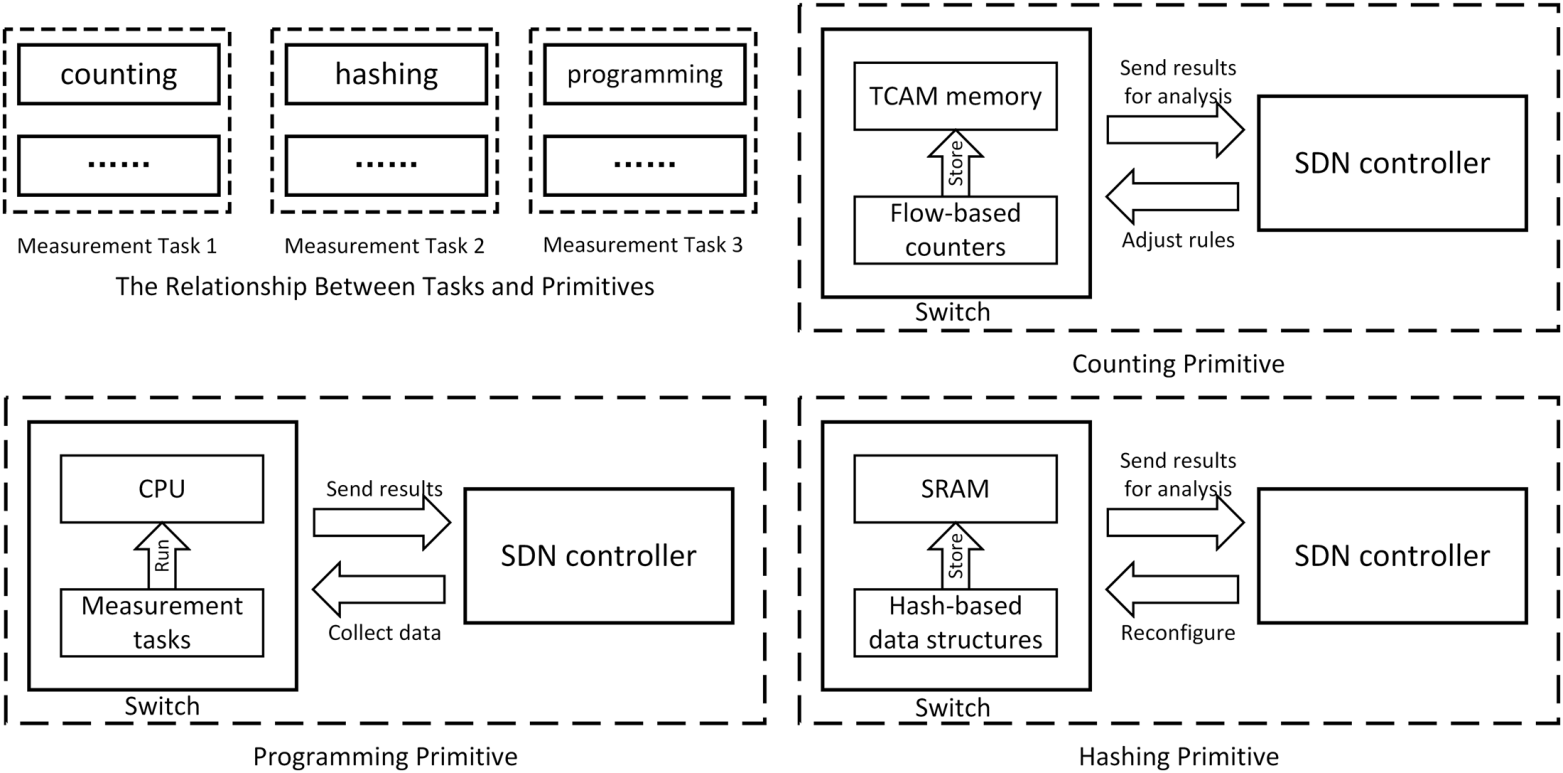
Resource Utilization. The resources consumed by different measurement primitives and the interactive process between switches and controllers.

SDN-enabled measurement techniques consist of several measurement primitives including counting, hashing, and programming [1–4]. As shown in Fig 1, these primitives consume various resources in switches and controllers including Ternary Content Addressable Memory (TCAM), processing time of CPUs, etc. Moreover, extra communication cost is introduced due to the bandwidth usage of the request and reply packets. Obviously, when there are hundreds of thousands of flows, collecting flow information may bring serious workload to the central controller.

To reduce the communication cost for collecting all the flow informations in SDN, one switch-based method called FlowCover [5] is proposed. FlowCover requests selected switches (referred as *polling switch*) to send the statistics of all flows on it. It should be guarantee that at least one polling switch is traversed by each flow. In this way, some request and reply packets of flow statistics are aggregated. However, this method may bring some duplicate pollings on the controller because the controller may poll same flows on different polling switches. The duplicate pollings bring extra resource consumption of switches and controller, such as more TCAM memory entries, and may result in the overuse of limited resources. For example, paper [6] shows the monitor rules of measurement require memory space, but the switch memory is limited as discussed in [7–10]. The TCAM memory is expensive (US$ 350 for a 1 M-bit chip) and power-hungry (about 15 Watt / 1 Mbit) as shown in [11]. Therefore, although switch-based method saves lots of communication cost, it may cause more resource stress on switches and controller than the flow-based method.

SDN offers the possibility of saving both the communication cost and resources of switches and controller by flexible routing of flows. With an appropriate flow routing, the number of required polling switches can be reduced and a flow will not be monitored for multiple times unless it is necessary. Accordingly, there are more opportunities to save the communication cost for collecting information without extra resource stress on the controllers by jointly optimizing flow routing and polling switch selection. As a common knowledge, changing the route may cause some problems such as it may break the load balance and longer routes may increase the total load of the network. The switch and link capacity can be applied to find a tradeoff between the optimization of the number of polling switches and the network load. The technical contributions of this paper can be summarized as follows:

- We formulate the joint optimization problem as an Integer Linear Programming (ILP) model and solve it in small-scale networks.
- We propose an algorithm to derive an optimal solution for multi-rooted tree topology which is commonly adopted in DCNs.
- We design an efficient heuristic algorithm to jointly optimize flow routing and polling switch selection on general topology.
- We conduct extensive simulations to evaluate the performance of the algorithms. It is found that up to 51.16% communication costs are saved on multi-rooted tree topologies compared with that of FlowCover, and up to 55.76% costs are saved on general topologies.

This paper is organized as follows. At first, some related works are discussed. Then the motivation of jointly optimizing flow routing and polling switch selection is presented. After that, the joint optimization problem is formulated as an ILP model and an optimal algorithm on multi-rooted tree topology is proposed. Then we design an efficient heuristic algorithm for the problem, followed by extensive simulation results. At last, the conclusion is given.

## Related Works

There are a lot of previous works focusing on how to measure the network resource utilization. Some of the works are leveraging the TCAM on switches to measure the flow information [1, 3], while some others design efficient software based schemes to monitor the traffic [4, 12, 13]. However, they all focus on the concrete technique to measure network resource utilization, but not on how to reduce the communication cost for collecting flow information.

As far as we know, FlowCover [5] is the only existing work focusing on how to optimize the cost for collecting flow information. Different from the traditional flow-based monitor scheme, such as NetFlow [14] and sFlow [15], FlowCover requests *all* the flow information on the selected switches instead of the information of a specific flow. In this way, FlowCover aggregates the information of multiple flows into a single packet and reduces the communication cost. However, FlowCover assumes that the flow route is fixed, and hence loses part of the optimization space and causes duplicate pollings. To the best of our knowledge, we are the first to optimize communication cost for collecting flow information in SDN by jointly optimizing flow routing and polling switch selection.

## Motivation

**Fig 2:**
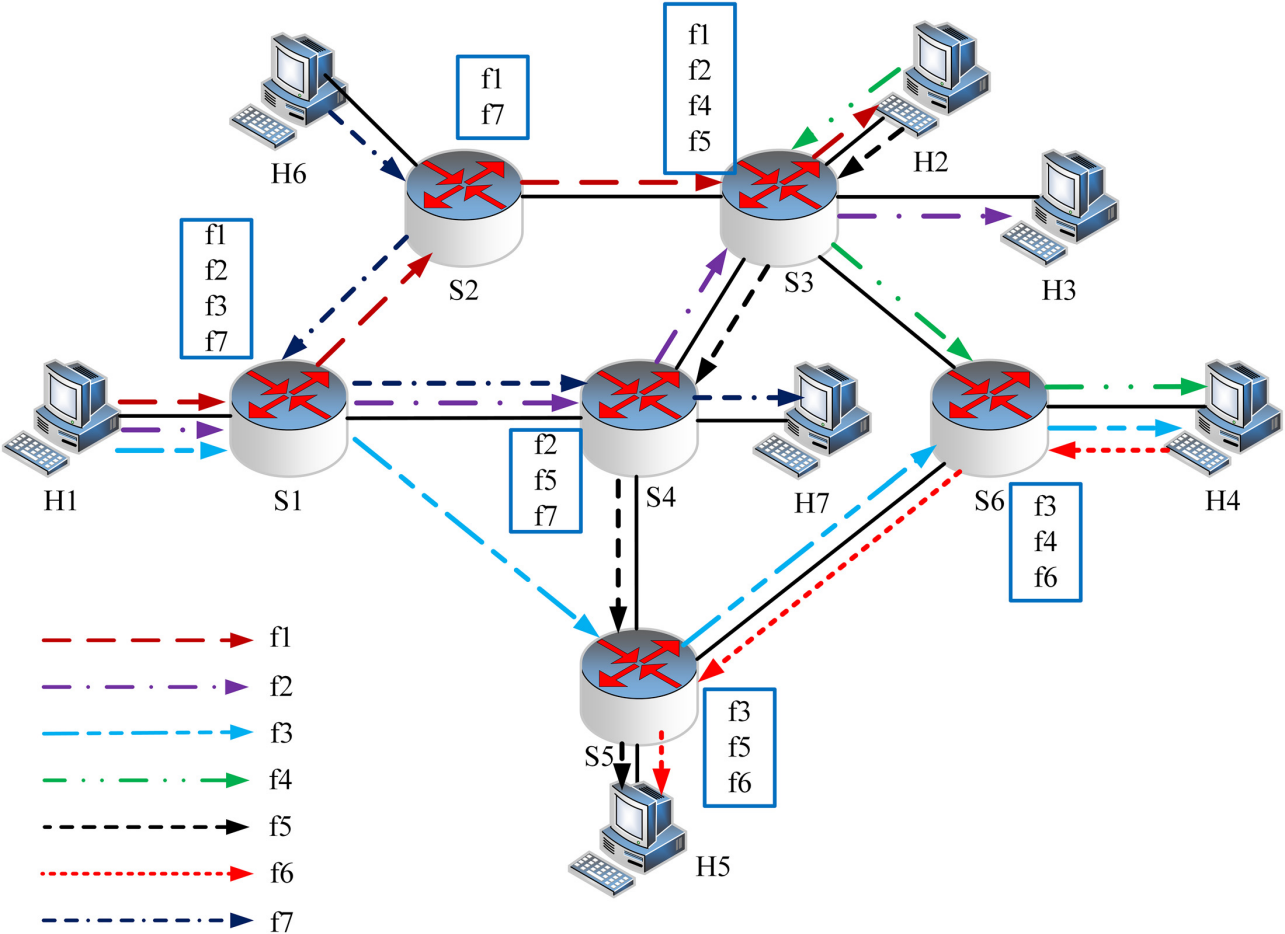
Motivation Example (a). The paths of flows obtained by shortest path algorithm. The optimal solution is polling the flow information on switches *S*_2_, *S*_3_ and *S*_6_.

**Fig 3:**
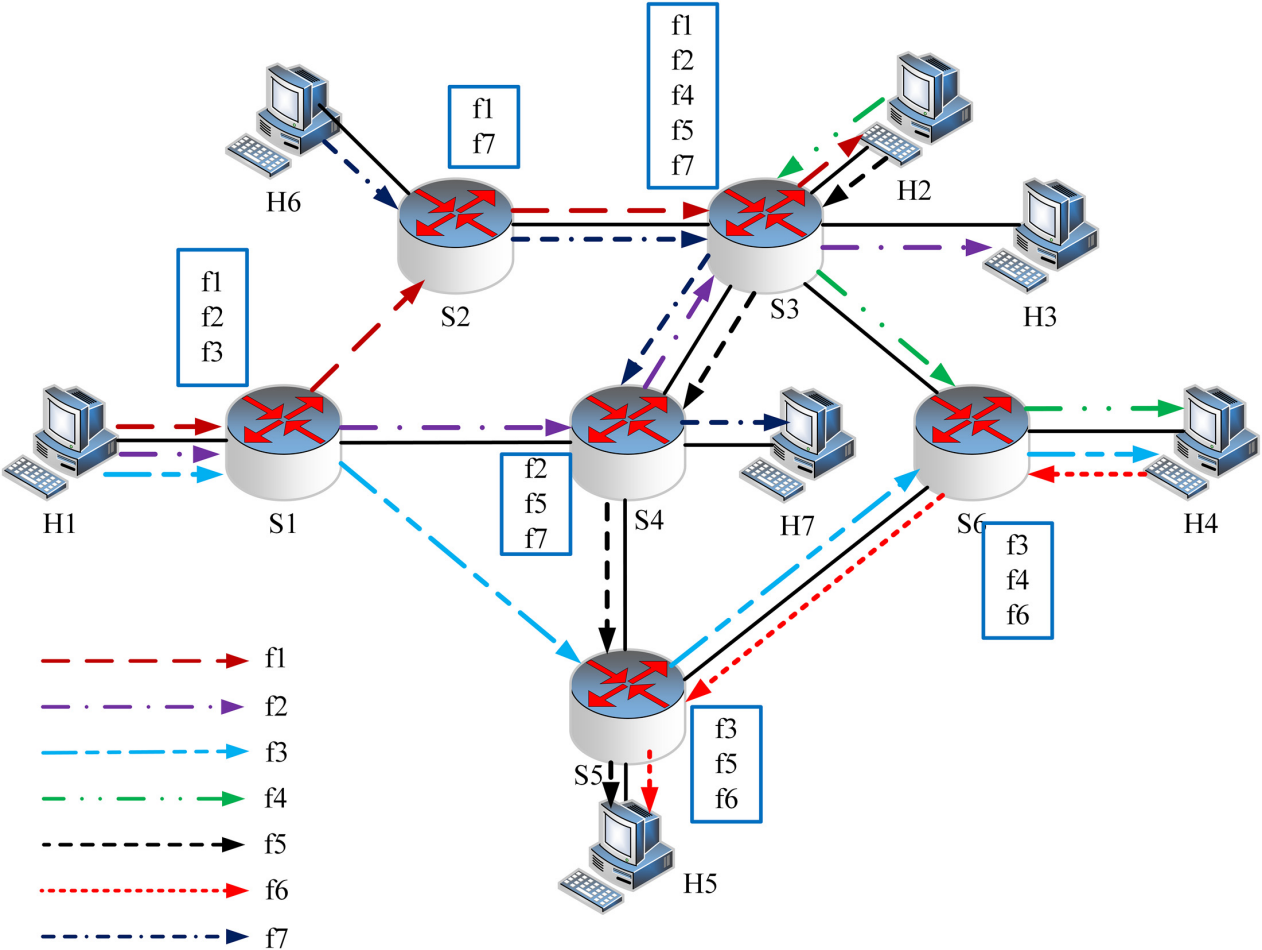
Motivation Example (b). The alternative shortest paths of flows with flexible routing. The optimal solution is polling the flow information on switches *S*_3_ and *S*_6_.

In this section, we present why our joint optimization scheme can help reducing the communication cost of flow measurement in SDNs through a motivation example. The example is shown in Figs 2 and 3. In this example, there are six switches and six hosts. Seven active flows are plotted in different colors and line styles. The source and destination nodes of each flow are given as *f*_1_: *H*_1_ − *H*_2_, *f*_2_: *H*1 − *H*3, *f*_3_: *H*_1_ − *H*4, *f*_4_: *H*_2_ − *H*_4_, *f*_5_: *H*_2_ − *H*_5_, *f*_6_: *H*_4_ − *H*_5_, *f*_7_: *H*_6_ − *H*_7_. Each of the switches only holds the partial view of all the flows. The partial view of each switch is given in the rectangles.

Considering that different measurement tasks require different types of resources (e.g. CPU, memory) and one task may require multiple resources, this paper uses communication cost which is proportional to the other resources utilization of switches and controller to show the performance of joint optimization scheme.

According to the OpenFlow specification [16], the message of an individual flow statistics request counts 56 bytes, and the header of a reply message counts 12 bytes, and the message body of reply message counts 96 bytes per flow. Taking the other packet headers (66 bytes per packet including headers of Ethernet, IP and TCP) into account, collecting the information of one flow requires 296 bytes data which is referred as *communication cost*.

In the example, the network carries seven flows. The flow-based scheme queries one of the switches along the route of each flow. In this case, 7 request messages as well as 7 reply messages will be generated and sent between the controller and switches. Hereby, there are 7 × (122 + 174) = 2072 bytes on wire for flow statistics.

FlowCover [5] selects polling switches to monitor all the flow information on them. However, the reducing of communication cost is limited by the fixed flow routing. When the flows are routed as shown in Fig 2 which is obtained by shortest path algorithm, the optimal solution of FlowCover is polling the flow information on switches *S*_2_, *S*_3_ and *S*_6_. There are (66 + 56) × 3 = 366 bytes on wire for request messages and (66 + 12) × 3 + 96 × 9 = 1098 bytes for reply messages (*f*_1_ is monitored twice on *S*_2_ and *S*_3_, and *f*_4_ is monitored twice on *S*_3_ and *S*_6_), so the communication cost is 1464 bytes, and about 29.34% communication cost on the flow information statistics is saved compare with that of flow-based scheme.

It should be pointed out that there are some changes of the length of request and reply messages in the latest OpenFlow specifications. However, the small differences will not lead to a fundamental change of the conclusion, and in order to make a fair comparison, we use the same version of OpenFlow specification with FlowCover [5].

If we change the route of flow *f*_7_ as shown in Fig 3 which is also the shortest path, the optimal solution of FlowCover is polling the flow information on switches *S*_3_ and *S*_6_. The communication cost is reduced to (66 + 56) × 2 + (66 + 12) × 2 + 96 × 8 = 1168 bytes (*f*_4_ is monitored twice on *S*_3_ and *S*_6_). It saves 43.63% communication cost compared with that of flow-based scheme. Meanwhile, it saves 20.22% less bytes compared with that of FlowCover if the routes can be changed. From above examples, we see that *jointly optimizing flow routing and polling switch selection can greatly reduce the communication cost on flow information statistics*.

## Methods

### Problem Formulation

In this section, the joint optimization problem is formulated as an Integer Linear Programming (ILP) model. All the notations used in this paper is shown in Table 1.

**Table 1:** Notations Used in This Paper.

Notation	Description
*V*	The set of nodes
*E*	The set of links
*F*	The set of flows
*f*_*sdt*_	The parameter denotes the *t* ^*th*^ flow from node *s* to node *d*.
$e_{sdt}^{uv}$	A binary variable indicates whether link (*u*, *v*) is in the path of flow *f*_*sdt*_
$q_{sdt}^{uv}$	A variable denotes the voltage value of link (*u*, *v*) in the path of flow *f*_*sdt*_
$z_{sdt}^{u}$	A binary variable indicates whether flow *f*_*sdt*_ goes through node *u*
*m*_*u*_	A binary variable indicates whether node *u* is a polling switch
$y_{sdt}^{u}$	A binary variable indicates whether the statistics of flow *f*_*sdt*_ can be collected from node *u*
*h*_*ij*_	The minimum hop number from node *i* to node *j*
*l*_*reqheader*_	The length of the request packet header
*l*_*reqmsg*_	The length of the request packet payload
*l*_*flow*_	The length of a single flow entry in the flow table
*C*_*u*_	The flow table capacity of node *u*
*C*_*uv*_	The capacity of link (*u*, *v*)

The topology of network is abstracted as an undirected graph *G* = (*V*, *E*). *V* denotes the set of nodes and *E* denotes the set of edges, edge (*i*, *j*) indicates that node *i* and *j* can directly communicate with each other.

Firstly, to calculate a route from source node *s* to destination node *d* of the flow *f*_*sdt*_, the flow conversation constraint should be satisfied, which is given by

$$\begin{array}{c} \sum_{(u,v)\in E}e_{sdt}^{uv}-\sum_{(u,v)\in E}e_{sdt}^{vu}=\left\{\begin{array}{cc} 1 & \;\;\;\;\;\text{if}\;u=s\\ -1 & \;\;\;\;\;\text{if}\;u=d\\ 0 & \;\;\;\;\;\text{otherwise} \end{array}\right.\\ \forall f\in F,\;\forall u\in V \end{array}$$ (1)

**Fig 4:**
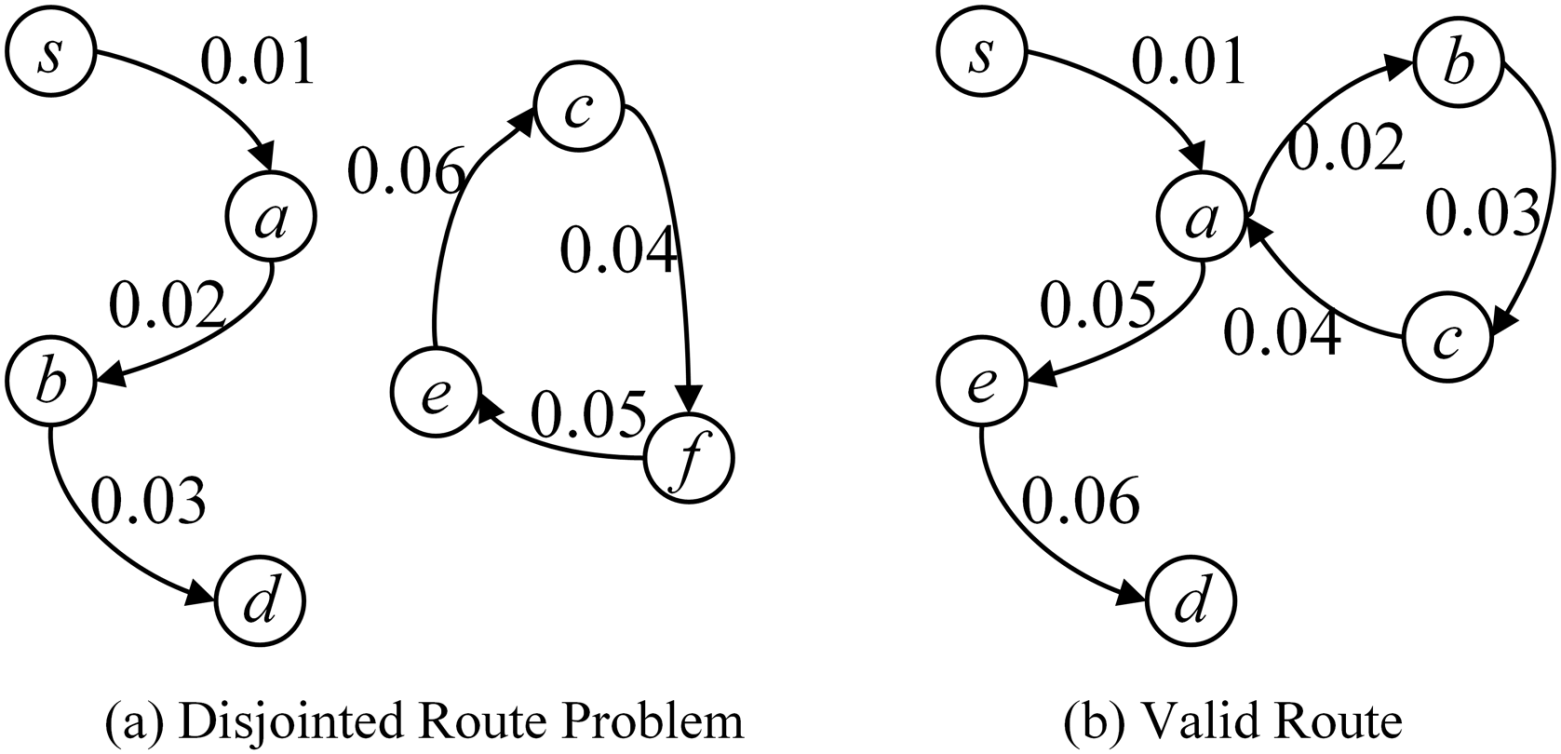
Voltage Constraint. The voltage constraint to solve the disjointed route problem and ensure the valid route in SDN.

However, the flow conversation constraint is not enough to ensure a valid route for each flow. For example, in Fig 4(a), the route of the flow between nodes *s* and *d* satisfies the flow conversation constraints, but it is an invalid route since the disjointed circle. To solve this problem, a positive voltage $q_{sdt}^{uv}$ is added to each link (*u*, *v*) if flow *f*_*sdt*_ goes through link (*u*, *v*), otherwise $q_{sdt}^{uv}$ is appointed to 0. For any node traversed by flow *f*_*sdt*_ except the destination node of flow *f*, the sum of the voltage values of its outbound links must be larger than that of its inbound links. This is called the *voltage constraint* [17]. The specific voltage value on each link is not important once the voltage constraint is satisfied.

It is notable that the non-simple path (i.e. path with circles) shown in Fig 4(b) is valid in SDN by configuring the flow tables in SDN switches. To ensure that the non-simple path routing also satisfy the voltage constraints, we formulate the voltage constraints as

$$q_{sdt}^{uv}\leq e_{sdt}^{uv}\;\;\;\forall f_{sdt}\in F,\;\forall \left(u,v\right)\in E$$ (2)

$$\begin{array}{c} \lambda \times z_{sdt}^{u}\leq \left\{\begin{array}{cc} 1+\sum_{(u,v)\in E}\left(q_{sdt}^{uv}-q_{sdt}^{vu}\right) & \text{if}\;u=d\\ \sum_{(u,v)\in E}\left(q_{sdt}^{uv}-q_{sdt}^{vu}\right) & \text{otherwise} \end{array}\right.\\ \;\forall u\in V,\;\forall f\in F \end{array}$$ (3)

*λ* is a positive constant number that is less than |*E*|^−1^. In Eq (3), the binary variable $z_{sdt}^{u}$ indicates whether node *u* is traversed by flow *f*_*sdt*_. Therefore, the following constraint must be satisfied

$$z_{sdt}^{u}\geq \frac{e_{sdt}^{uv}+e_{sdt}^{vu}}{2}\;\;\;\;\;\forall u\in V,\;\forall \left(u,v\right)\in E$$ (4)

When the routes are obtained for each flow, the next step is to select a polling switch for each flow. Let the binary variable *m*_*u*_ represent whether node *u* is a polling switch, and let the binary variable $y_{sdt}^{u}$ denote whether the statistics of flow *f*_*sdt*_ is collected on node *u*. Obviously, if node *u* is a polling switch and traversed by flow *f*_*sdt*_, the statistics of flow *f*_*sdt*_ can be collected from node *u*, i.e.,

$$\begin{array}{c} \frac{z_{sdt}^{u}+m_{u}-1}{2}\leq y_{sdt}^{u}\leq \frac{z_{sdt}^{u}+m_{u}}{2}\;\;\;\forall u\in V,\;\forall f\in F \end{array}$$ (5)

To collect the statistics of all flows in the network, each flow should traverse at least one polling switch, which is given by

$$\sum_{u\in V}y_{sdt}^{u}\geq 1\;\;\;\;\forall f\in F$$ (6)

At last, in order to avoid the possible flow congestion in switches, the following capacity constraints should be satisfied.

$$\sum_{f_{sdt}\in F}z_{sdt}^{u}\leq C_{u}\;\;\;\forall u\in V$$ (7)

$$\sum_{f_{sdt}\in F}e_{sdt}^{uv}\leq C_{uv}\;\;\;\forall \left(u,v\right)\in E$$ (8)

The objective of the joint optimization problem is minimizing the communication cost for collecting flow information which consists of request cost and reply cost. The request cost is determined by the number of polling switches. Let *l*_*reqheader*_ denote the length of the request packet header, and let *l*_*reqmsg*_ denote the length of request packet payload, hence the request cost is

$$\left(l_{reqheader}+l_{reqmsg}\right)\times \sum_{u\in V}m_{u}$$

Let *l*_*replyheader*_ denote the length of the reply packet header, and let *l*_*flow*_ denote the length of single flow entry, hence the replying cost is

$$l_{replyheader}\times \sum_{u\in V}m_{u}+l_{flow}\times \sum_{u\in V}\sum_{f_{sdt}\in F}y_{sdt}^{u}$$

As mentioned in Section, the OpenFlow specification 1.0 [16] is used in this paper. According to this specification, *l*_*reqheader*_ = 66 bytes, *l*_*reqmsg*_ = 56 bytes, *l*_*replyheader*_ = 78 bytes and *l*_*flow*_ = 96 bytes, Therefore, the objective of the problem is

$$\text{minimize}\;\;\;200\times \sum_{u\in V}m_{u}+96\times \sum_{u\in V}\sum_{f_{sdt}\in F}y_{sdt}^{u}$$ (9)

#### Complexity Analysis

The ILP model has (2|*E*| + 2|*V*|)|*F*| + 2|*V*| + |*E*| variables and (|*E*| + 4|*V*| + 1)|*F*| + (|*V*| + 1)|*E*| + |*V*| constraints. Similarity to the analysis of the complexity of algorithms, we can only take the orders of growth into account. Moreover, the number of edges in a graph is usually much greater than that of nodes, and the number of flows is also much larger than the number of edges. Thus, the number of variables and constraints in the ILP model is proportional to O(|E||F|).

### Offline Algorithm on Multi-Rooted Tree Topologies

The multi-rooted tree topology is widely adopted in Data Center Network (DCN) which is a popular instance of SDN. In DCN, no circle is allowed in the path of any flows, and each flow should be routed to one of the shortest paths between its two ends.

Accordingly, one more constraint should be added into the ILP model formulated in last section.

$$\sum_{(u,v)\in E}e_{sdt}^{uv}\leq h_{sd}\;\;\;\;\forall f\in F$$ (10)

where *h*_*sd*_ denotes the minimum hop number from the source node *s* to the destination node *d*.

**Fig 5:**
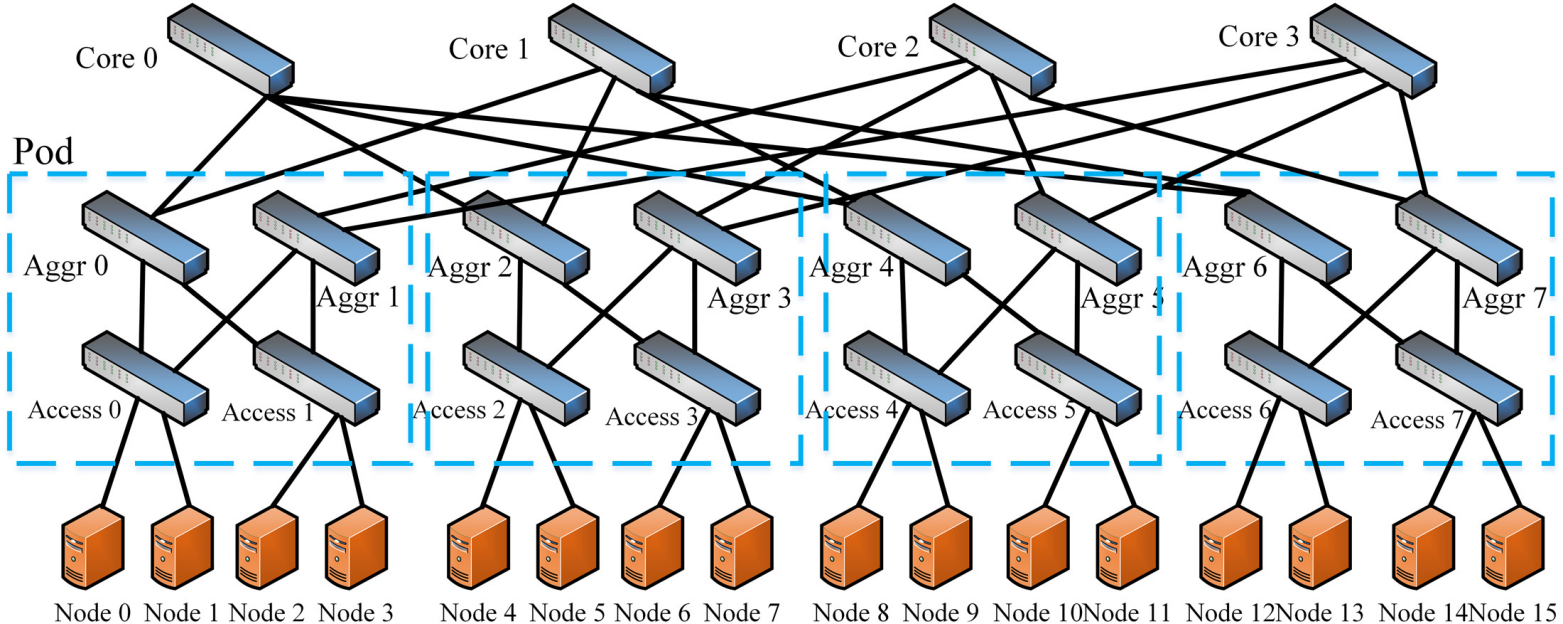
The PortLand Topology. The Portland topology with *k* = 4. The parameter *k* is the number of switch port which is the only factor that determines the network size of Portland.

It is believed that ILP model is usually intractable in large-scale networks. To verify this, we conduct several simulations to evaluate the solve elapsed time of the ILP model. The simulations are carried on PortLand [18] which is a type of multi-rooted tree topology in DCN. The parameter *k* is the number of switch port which determines the network size of PortLand. The PortLand topology with *k* = 4 is shown in Fig 5. The ILP model are solved by CPLEX [19] on a workstation with 2 Six-Core 2.00 GHz Intel Xeon E5-2620 CPUs and 16 GB memory. The results are shown in Table 2.

**Table 2:** Execution Time of Solving the ILP Model.

Number of Flows	k		
	4	6	8
8	4.836 s	57.876 s	1315.12 s
16	8.97 s	218.416 s	No solution found in 12 hours
32	20.873 s	9754.13 s	No solution found in 12 hours

According to Table 2, the execution time increases rapidly when the network size increases. It can be found that the execution time of solving the ILP model with 32 flows in a k = 6 multi-rooted tree topology is more than 2 hours. Meanwhile, we cannot find a feasible solution for the ILP model within hours when there are more than 16 flows on a k = 8 multi-rooted tree topology.

Since it is hard to solve the ILP model in large-scale networks, we design an optimal algorithm for this problem on multi-rooted tree topology. To prove the correctness of our algorithm, we not only present a solid theorem, but also compare the solution of ILP with that of the optimal algorithm on small size multi-rooted tree topology which is shown in S1 Appendix.

**Algorithm 1:** Minimizing Communication Cost on Multi-rooted Tree

**Require:** Network topology, flow set ***F***_1_, ***F***_2_, ***F***_3_

**Ensure:** Flow route ***R*** = {*r*_*f*_}, polling switch set ***P***

 1: **for** flow *f* ∈ ***F***_1_ **do**

 2:  *f*’s route *r*_*f*_ is uniquely determined, ***R***_***F***_ ← ***R***_***F***_ ∪ *r*_*f*_

 3:  *u* is the access switch traversed by *f*, ***P*** ← ***P*** ∪ *u*

 4:  Update *U*_*l*_ and *U*_*u*_

 5: **end for**

 6: Get ***T*** ⊂ ***F***_2_ ∪ ***F***_3_ such that *f* ∈ ***T*** ends at a host *a* ∈ ***P***

 7: ***F***_2_ ← ***F***_2_ − ***T***, ***F***_3_ ← ***F***_3_ − ***T***

 8: **for** flow *f* ∈ ***F***_2_ **do**

 9:  Determine *r*_*f*_ by 1) using the aggregate switch *u* ∈ ***P*** with enough remaining capacity; 2) using the aggregate switch *u* with maximum enough remaining capacity, ***P*** ← ***P*** ∪ *u*

 10:  ***R*** ← ***R*** ∪ *r*_*f*_ and update *U*_*l*_ and *U*_*u*_

 11: **end for**

 12: **for** flow *f* ∈ ***F***_3_ **do**

 13:  Determine *r*_*f*_ by 1) using the path with minimum, but at least one switch *a* ∈ ***P***; 2) using aggregate switches with minimal enough remaining capacity and core switch with maximum enough remaining capacity *c* ∉ ***P***

 14:  ***R*** ← ***R*** ∪ *r*_*f*_ and update *U*_*l*_, *U*_*u*_

 15: **end for**

 16: Route flows in ***T*** through least switches in ***P***

 17: **return** ***R*** and ***P***

In this algorithm, ***F***_1_ is the set of flows within an access switch (i.e. the source and destination node are connected to the same access switch), ***F***_2_ is the set of the flow whose source and destination are connected to different access switches but within the same pod (*pod* is a group of access and aggregate switches as shown in Fig 5). ***F***_3_ is the set of cross-pod flows. In addition, *U*_*l*_ and *U*_*u*_ is used to denote the used capacity of link *l* and node *u*, respectively. ***P*** is the set of polling switches, ***T*** is the set of flows whose route is not determined but the access switch of the flow is already a polling switch.

For any unrouted flow in ***F*** **1**, there is only one candidate shortest path from source to destination through the access switch. So that the access switch must be selected as a polling switch (Line 1–5). The statistics of any other unrouted flows originating or ending at the hosts connected to this access switch will also be collected since the access switch is already selected as polling switch. Such unrouted flows will be put into set ***T*** as in Line 6–7.

For any unrouted flow in ***F*** **2**, the candidate shortest paths may traverse different aggregate switches. To minimize the number of polling switches, the routes of the flows are packed together. In other words, as long as there exists enough resource in the aggregate switches, the flows will be routed through the same aggregate switche which is already selected as a polling switch (Line 8–11).

While choosing the routes for the unrouted flows in ***F*** **3**, the candidate shortest path traversing only one polling switch is preferred. If there is no any polling switch along its all candidate paths, one core switch must be selected as polling switch. In such case, to minimize the number of polling switches, the routes of the flows are packed together (Line 12–15) which means the flows will be routed through the same core switch if the switch is already a polling switch.

Finally, for the unrouted flows in ***T***, the candidate shortest path traversing minimum number of polling switches is preferred (Line 16).

For Algorithm 1, we have the following theorem:

**Theorem 1.** *The Algorithm 1 can derive an optimal routing and polling switch selection scheme to minimize the communication cost for collecting all the flow information on multi-rooted tree topology*.

Proof: It is obvious that in Line 1–11 of Algorithm 1, the algorithm uses the minimum polling switches to monitor the flow information and each flow traverses only one polling switch. Therefore, the optimal route of the flows that are originating and ending under the same access switch or aggregate switch can be obtained. As in Line 8–11, the algorithm selects the necessary polling switches and each flow traverses only one polling switch. In Line 12–15, we do not increase the number of polling switches and each flow traverses only one or two (when the resources of the aggregate switches are not enough) polling switches, thus the communication cost is also minimized. At last, the remaining flows, all of whose routes are monitored by at least one polling switch, are routed to the path traverse minimum polling switches which means the communication cost can be also minimized in this step. In summary, Algorithm 1 can guarantee its solution is optimal at every step, and hence it derives the optimal solution.

#### Complexity Analysis

As shown in Algorithm 1, we need to find the route of three types of flows denotes by ***F*** **1**, ***F*** **2** and ***F*** **3**. Algorithm 1 can determine the route of each flow according to the existing polling switches. Thus, the computational complexity of Algorithm 1 is O(|F|) (***F*** denotes the set of all flows).

### Offline Algorithm in General Topology

In this section, a heuristic algorithm is proposed to optimize the communication cost on general topologies. In general topologies, it is believed that the more a switch contributes to the network connectivity, the more flows the switch will carry. Therefore, the switch has more possibility to be chosen as a polling switch. Accordingly, we first identify the contribution of each switch to the network connectivity, then route the flows in the network through minimum number of polling switches. Following this line of thought, how to determine the switch importance is introduced firstly, and an algorithm to jointly optimize flow routing and polling switch selection is presented.

**Algorithm 2:** Calculate Switch Importance

**Require:** Importance spread matrix *M*

**Ensure:** Switch importance vector *π*

 1: Initialize $\pi^{\left(0\right)}\leftarrow \frac{1}{S}1$

 2: *π* ^(1)^ ← *π* ^(0)^ *M*, *k* ← 1

 3: **while** |*π* ^(*k*)^ − *π* ^(*k* − 1)^| > *ϵ* **do**

 4:  *π* ^(*k*+1)^ ← *π* ^(*k*)^ *M*

 5:  *k* ← *k* + 1

 6: **end while**

 7: **return** *π* ^(*k*)^

#### Switch Importance Value Calculation

It is widely believed that the switches contribute more to the network connectivity may be traversed by more flows. Thus, the switches may have higher potential to be selected as a polling switch since they can carry more flows. Meanwhile, the communication cost can be reduced by aggregating the flow information into fewer reply packets. To identify each switch’s contribution to the network connectivity, the *importance value* for each switch is used.

There are many ways to determine the importance value of each switch [20]. In method we used, each switch evenly spreads its importance to its adjacent switches and receives importance from its adjacent switches. At the equilibrium state, the switches with higher importance may be the ones which contribute more to the network connectivity. Suppose there are *S* switches, to calculate such importance value of each switch, a matrix *M*_*S* × *S*_ is constructed as

$$m_{ij}=\left\{\begin{array}{cc} \frac{1}{D_{i}} & \text{if}\;\text{switch}\;i\;\text{and}\;j\;\text{are}\;\text{connected}\\ 0 & \text{otherwise} \end{array}\right.$$ (11)

where *D*_*i*_ is the nodal degree of switch *i*, and let *π* be an 1 × *S* vector denotes the importance of *S* switches. The switch importance can be calculated by solving the equation group

π=πM (12)

Since there are infinite numbers of solutions for Eq (12), and we only focus on the relative importance of the switches, we suppose the sum of the importance values is 1, i.e.

π1=1 (13)

where **1** is a *S* × 1 vector with all items being 1. By solving the equation group formed by Eqs (12) and (13), we can get the importance value of each switch.

It is worth noting that this equation group is difficult to solve when matrix *M* has a large size. Therefore, we need an efficient algorithm to solve it, the procedure of calculating the importance value is shown in Algorithm 2.

**Algorithm 3:** Jointly optimizing flow routing and polling switch selection

**Require:** Network topology and flow set ***F***

**Ensure:** Flow route {***R***_***f***_} and polling switch set ***P***

 1: Get switch importance value vector *π* by using Algorithm 3 (without loss of generality, we assume *π*_1_ ≥ *π*_2_ ≥ ⋯ ≥ *π*_*S*_)

 2: *U*_*i*_ denotes the used capacity of switch *i*, *C*_*i*_ denotes the total capacity of switch *i*.

 3: **for** *i* = 1 to *S* **do**

 4:  ***P*** ← ***P***∪{*i*}

 5:  **if** switch *i* carried some flows whose polling switches are not *i* **then**

 6:   try to find a new path for flow *f*, where the new path must traverse its polling switch and does not traverse switch *i*, then update the route *R*_*f*_

 7:  **end if**

 8:  **while** *U*_*i*_ < *C*_*i*_ **do**

 9:   Find a flow *f* ∈ ***F*** traverses minimum polling switches such that 1) it traverses switch *i*; 2) The route has minimum hops

 10:   **if** find a flow *f* ∈ ***F*** in Line 9 **then**

 11:    Set the *R*_*f*_ of the selected flow, ***F*** ← ***F*** − *f*

 12:    **continue**

 13:   **end if**

 14:   **break**

 15:  **end while**

 16: **end for**

 17: **return** {*R*_*f*_} and ***P***

#### Joint Optimization Algorithm on General Topology

Given the importance value of each switch, the switches with larger importance value can be chosen to be polling switches. Then the flows can be routed to the polling switch for minimizing communication cost. Following this line of thought, we propose Algorithm 4 to joint optimizing flow routing and polling switch selection on general topologies.

Firstly, let the switch with largest importance value be a new polling switch (Say it is switch *i*, line 4). If there are already some flows in switch *i*, these flows should be rerouted. For example, if switch *i* is traversed by the route of flow *f*, and the polling switch of flow *f* is *j* instead of *i*, flow *f* should be rerouted to avoid switch *i* and traverse switch *j* since the routes of flows should traverse minimum number of polling switches (Line 6).

For each unrouted flow, one minimum-hop path passing through switch i is chosen as its candidate path. The smaller the number of polling switches along its candidate path is, the earlier the flow will be routed. The numbers of hops of multiple candidate paths are used to break the tie (Line 9–15). A flow will be successfully routed as long as the resources along the its candidate path could be successfully allocated.

#### Complexity Analysis

The main loop of Algorithm 3 iterates for O(|S|) times. In the loop, the worst case is that the algorithm needs to find the candidate path for every flow. Therefore, the computational complexity of Algorithm 3 is O(|F||S|).

## Simulation Results

In this section, the performance of algorithms is studied through extensive simulations. The performance of algorithms on multi-rooted tree topologies and general topologies is evaluated.

As a benchmark, the performance of FlowCover is also tested. In addition, all the switch capacity constraint only works in our algorithm but not on FlowCover scheme, since the flow routing of FlowCover is fixed.

As mentioned in Section, it is hard to calculate the various resource utilization accurately, so the communication cost which is proportional to the resource utilization is used to evaluate the algorithm performance.

### Simulation Setup

Firstly, we introduce the input parameters (e.g., network topologies, traffic datasets) and the hardware environment used in the simulations.

#### Network Topologies

Though there are many multi-rooted tree topologies, such as PortLand [18] and VL2 [21], the simulations are carried on the PortLand which is commonly used in DCNs. The only parameter that affects the network size of PortLand is *k* which denotes the number of switch ports. The performance of Algorithm 1 is evaluated with different parameter *k*. The topology with *k* = 4 is shown in Fig 5.

**Fig 6:**
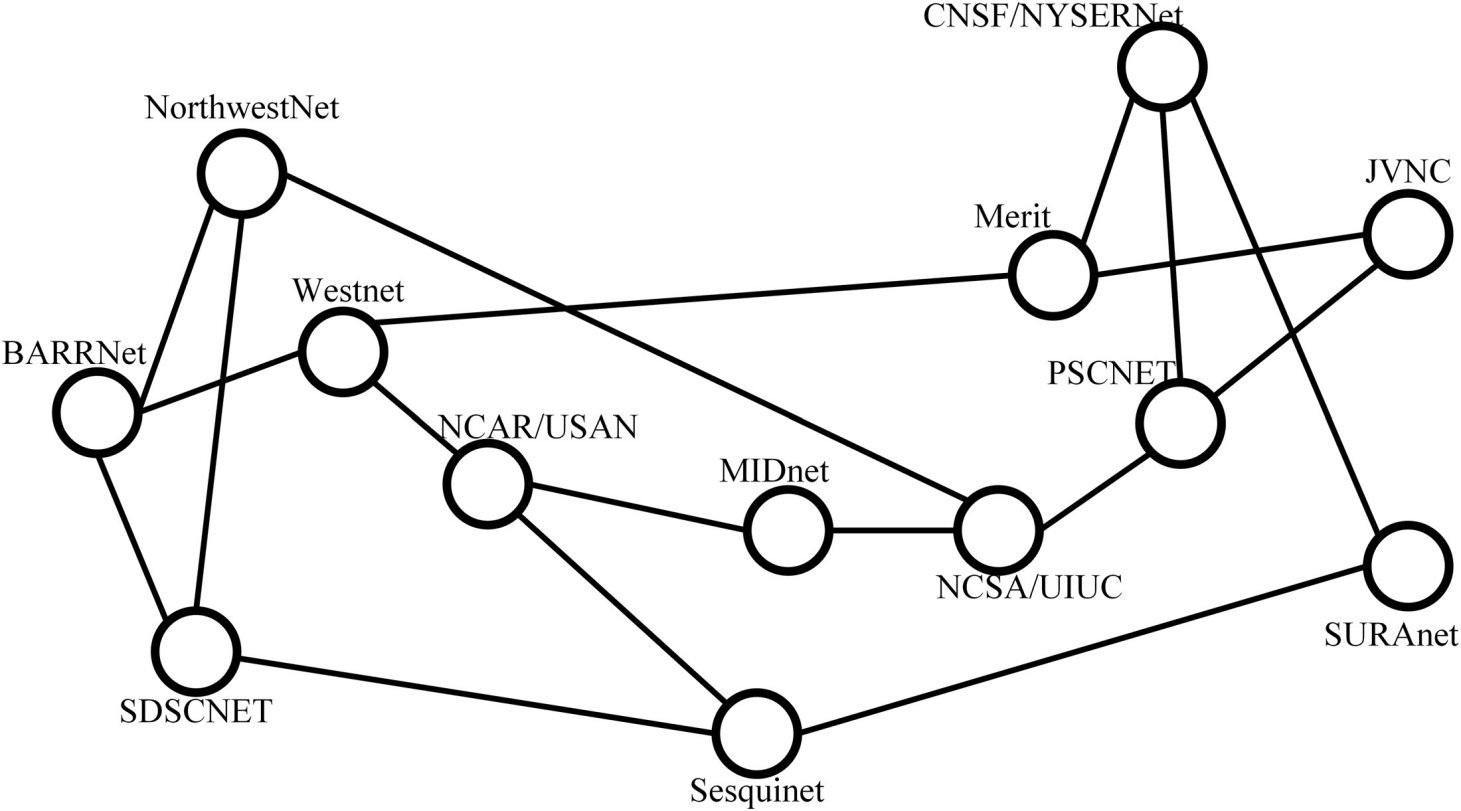
NSFNET. The NSFNET topology which includes 13 nodes and 18 links.

Besides the multi-rooted tree topologies, the simulations are also carried on two types of general topologies. First is the NSFNET [22] which is shown in Fig 6. Since the number of nodes and links in NSFNET is fixed, when we evaluate the performance of algorithms with different number of nodes, we use the random topologies with power law degree distribution generated by the NetworkX [23] which is a well-known Python Library used in network simulations.

#### Traffic Datasets

In multi-rooted tree topologies, the flows start and end in the hosts. Considering that in DCN, each host has the same possibility to send a request. Thus, we randomly generate the source and destination hosts of flows with uniform distribution and ensure that every host has at least one flow.

In general topologies, the source and destination nodes of flows are generated with Poisson distribution which is widely used in traffic matrix estimation [24]. The number of flows is changed in different scenarios.

All the simulations are run on a workstation with 2 Six-Core 2.00 GHz Intel Xeon E5-2620 CPUs and 16 GB memory.

### Performance on Multi-rooted Tree

In this subsection, the performance of Algorithm 1 on multi-rooted tree is studied. At first we compare the result of Algorithm 1 with that of ILP model in small-scale networks, then the case that no capacity constraint enforced into the network is studied to see the lower bound of the algorithm performance. After that, the impact of flow capacity on the communication cost is evaluated.

#### Comparison with the Result of ILP Model

Since the ILP model is intractable in large-scale networks, the simulations are carried on a PortLand topology with the parameter *k* = 4. To make the detail solution of ILP model and Algorithm 1 clear, we generate eight bidirectional flows, and the distribution of the flows can be found in S1 Appendix. By applying both ILP model and Algorithm 1 on this scenario, we get the results shown in Table 3. The ILP model is solved by CPLEX [19], and the detail results can be found in S1 Appendix.

**Table 3:** Result of ILP Model and Algorithm 1.

Algorithm	Switch Capacity	Communication Cost
ILP	0	1168
Algorithm 1	0	1168
ILP	3	1368
Algorithm 1	3	1368

The solution derived by ILP model and Algorithm 1 are different in terms of the flow routing and polling switch selection. However, according to the results from Table 3, we can see that the results of Algorithm 1 are exactly the same as the optimal results derived by ILP model in terms of the communication cost. It certificates the correctness of the Algorithm 1 and its optimality proof.

#### Performance with Unlimited Flow Table Capacity

Assuming the capacity of switches is unlimited, we study the performance of Algorithm 1 from two perspectives. First is how the performance change with the network size, and the second is how the performance impacted by the number of flows. For the first perspective, we set the flow number to be 512 and change the PortLand parameter *k* (the only factor determining the network size) to evaluate the algorithm performance. For the second perspective, we set the PortLand parameter *k* to be 16 and put different numbers of flows into the network to evaluate the communication cost for collecting flow information. The detail results of the simulations can be found in S2 Appendix.

**Fig 7:**
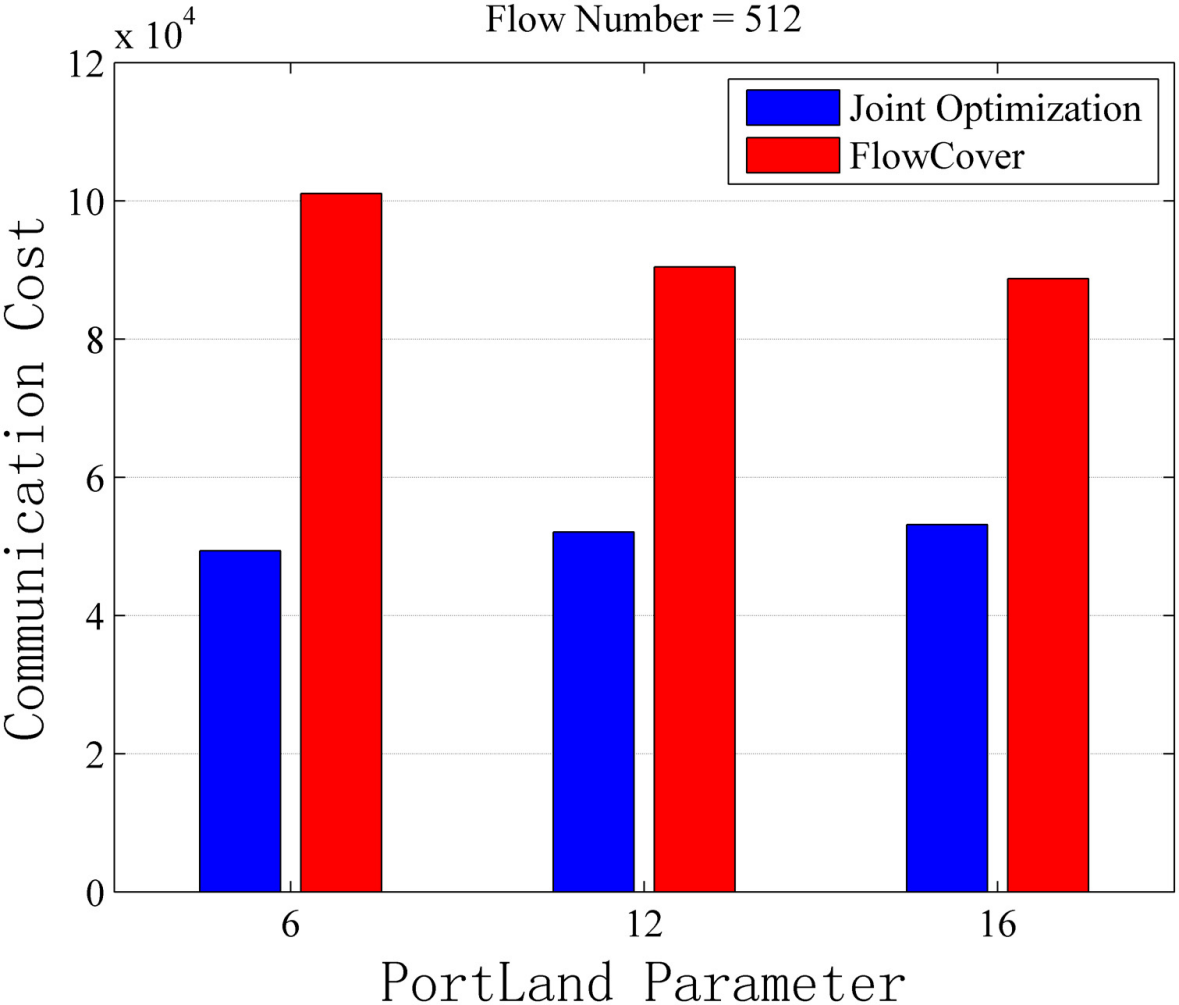
Communication Cost vs. Portland Parameter with No Capacity Constraint. The simulation result with 512 flows and different network size. The flow table capacity is unlimited in this scenario.

**Fig 8:**
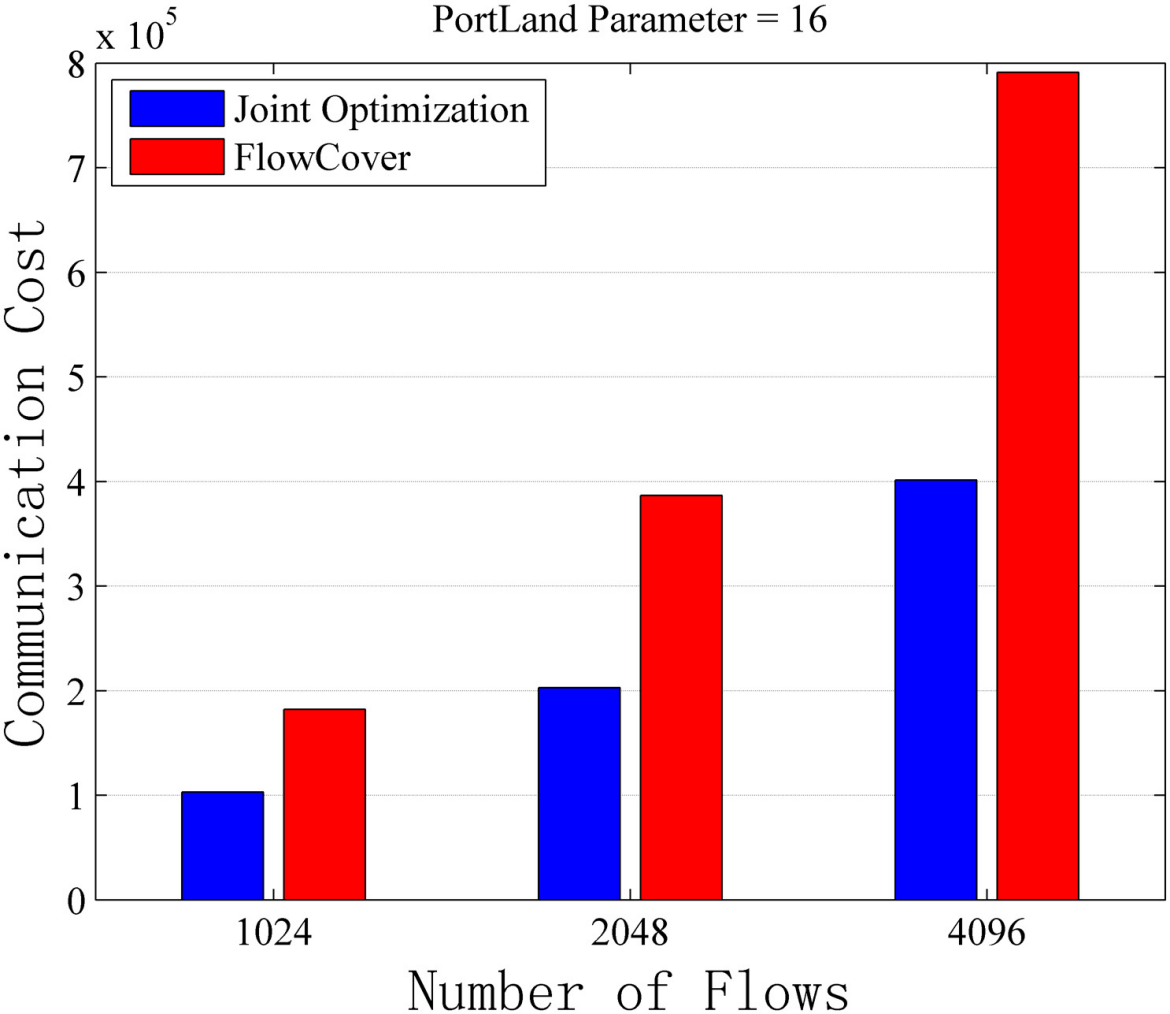
Communication Cost vs. Number of Flows with No Capacity Constraint. The simulation result with different number of flows on a *k* = 16 PortLand topology. The flow table capacity is unlimited in this scenario.

Fig 7 shows joint optimization algorithm can save 40.10%–51.16% communication cost with difference network size. Fig 8 shows joint optimization algorithm brings 43.50%–49.28% performance improvement with different numbers of flows.

According to the routing principles of DCNs, the routes of flows must be the shortest path in the multi-root tree. Therefore, if the topology is fixed, the communication cost of either joint optimization or FlowCover is only slightly affected by the network size from Fig 7. Meanwhile, the communication cost of joint optimization is reduced by routing optimization compared with that of FlowCover.

On the other hand, the communication cost increases when the flow number in the network increases (see Fig 8) since monitoring more flows requires more communication cost. Meanwhile, the joint optimization scheme always saves about half of the communication cost no matter how many flows in the networks compared with that of FlowCover.

#### Performance with Limited Flow Table Capacity

To study how the performance of joint optimization impacted by the switch capacity, we set the PortLand parameter *k* to be 16 and evaluate how the communication cost changes with the different flow capacities and different numbers of flows in the network. The detail results of the simulations can be found in S2 Appendix.

**Fig 9:**
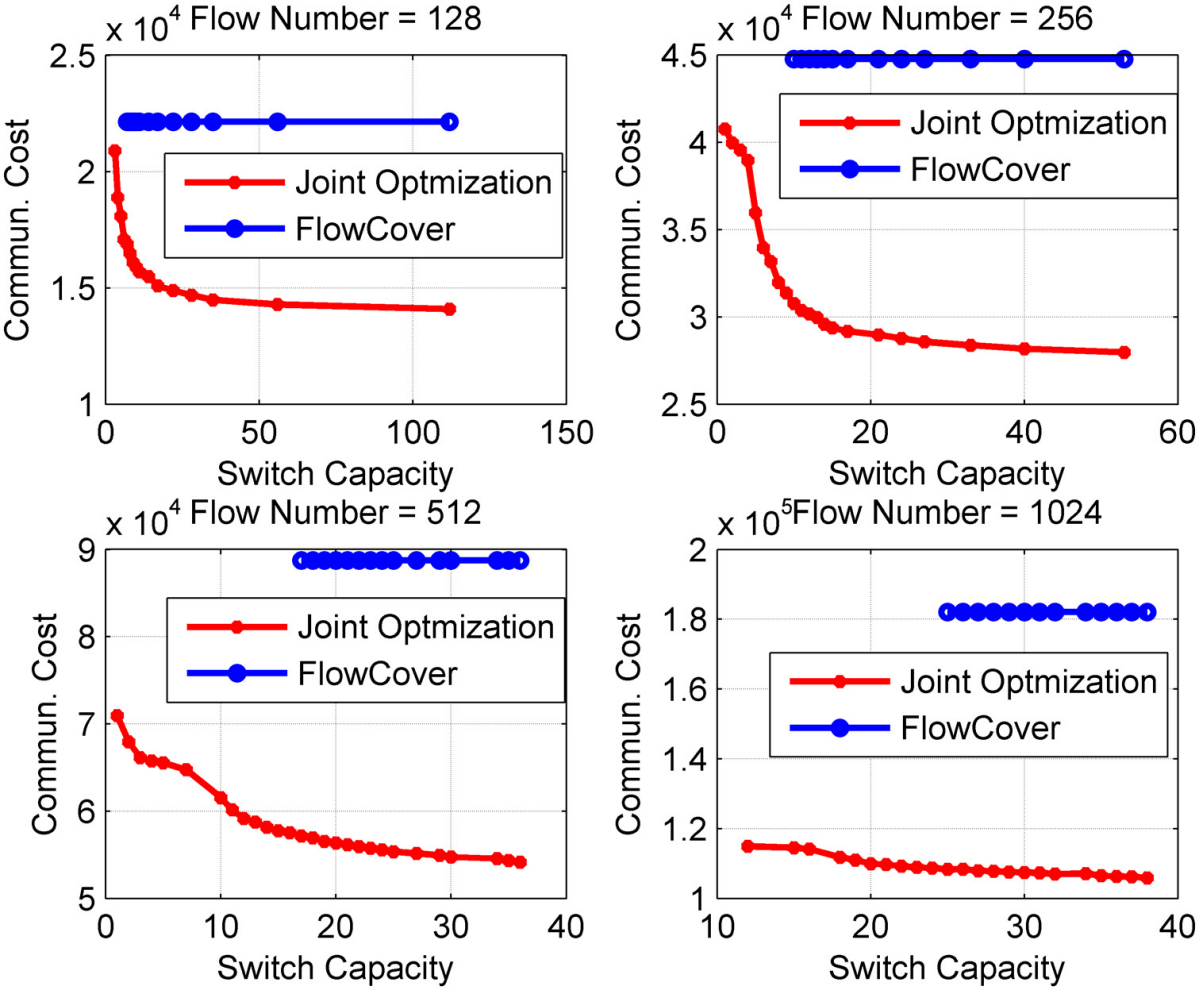
Communication Cost on PortLand vs. Switch Capacity. The simulation result with different switch capacities. The number of flows is set to a value in range of 128 to 1024. The Portland parameter *k* is 16.

Simulation results are shown in Fig 9. There are some obvious results, such as the communication cost decreases when the switch capacity increases, and joint optimization has more advantages when there are more flows. Additionally, we can get some other results.

Even if a very small flow capacity is set, joint optimization method can obtain a solution with lower communication cost than that required by FlowCover scheme. On the one hand, the uniform distribution of flows in the network increases the required number of polling switches. Accordingly, joint optimization scheme has the chance to optimize both load balance and communication cost. If there are more flows in the network, the larger probability flow congestion occurs. This is why we get a better load balance without pay much communication cost when there are more flows in the network.

### Performance on General Topology

In this subsection, we study the performance of our algorithm on general topology. At first we compare the result of our algorithm with that of the ILP model in NSFNET topology. After that, the performance of algorithm with unlimited and limited flow capacity is evaluated in different size topologies with power law degree distribution.

#### Comparison with the Result of ILP Model

As mentioned above, the ILP is intractable in large-scale networks, thus the simulations are carried on the NSFNET shown in Fig 6. By applying Algorithm 3 and ILP model on the NSFNET topology with small number of flows, we can get the result in Table 4. The distribution of the flows can be found in S3 Appendix. In the simulations, we present the result with unlimited flow table capacity and limited capacity. The capacity is set to 3 in the limited capacity scenarios.

**Table 4:** Result of ILP Model and Algorithm 3.

Number of Flows	Unlimited Capacity		Limited Capacity	
	ILP Model	Algorithm 3	ILP Model	Algorithm 3
5	680	680	880	880
8	968	968	1368	1464
10	1160	1160	1760	1856

From Table 4, it can be found that Algorithm 3 can get the optimal result in the scenarios presented with unlimited capacity. Meanwhile, in limited capacity scenarios, Algorithm 3 can get near optimal results, the gap between the results of Algorithm 3 and the optimal solution is less than 7%.

To evaluate the performance improvement in larger network, we present the result of Algorithm 3 in following paragraphs. As a benchmark, FlowCover [5] is also evaluated.

#### Performance with Unlimited Flow Table Capacity

In this subsection, we study the performance of our algorithm on general topology. To this end, different size topologies with power law degree distribution are generated. The result of Algorithm 3 with unlimited and limited flow capacity is evaluated. The detail results of the simulations can be found in S4 Appendix.

**Fig 10:**
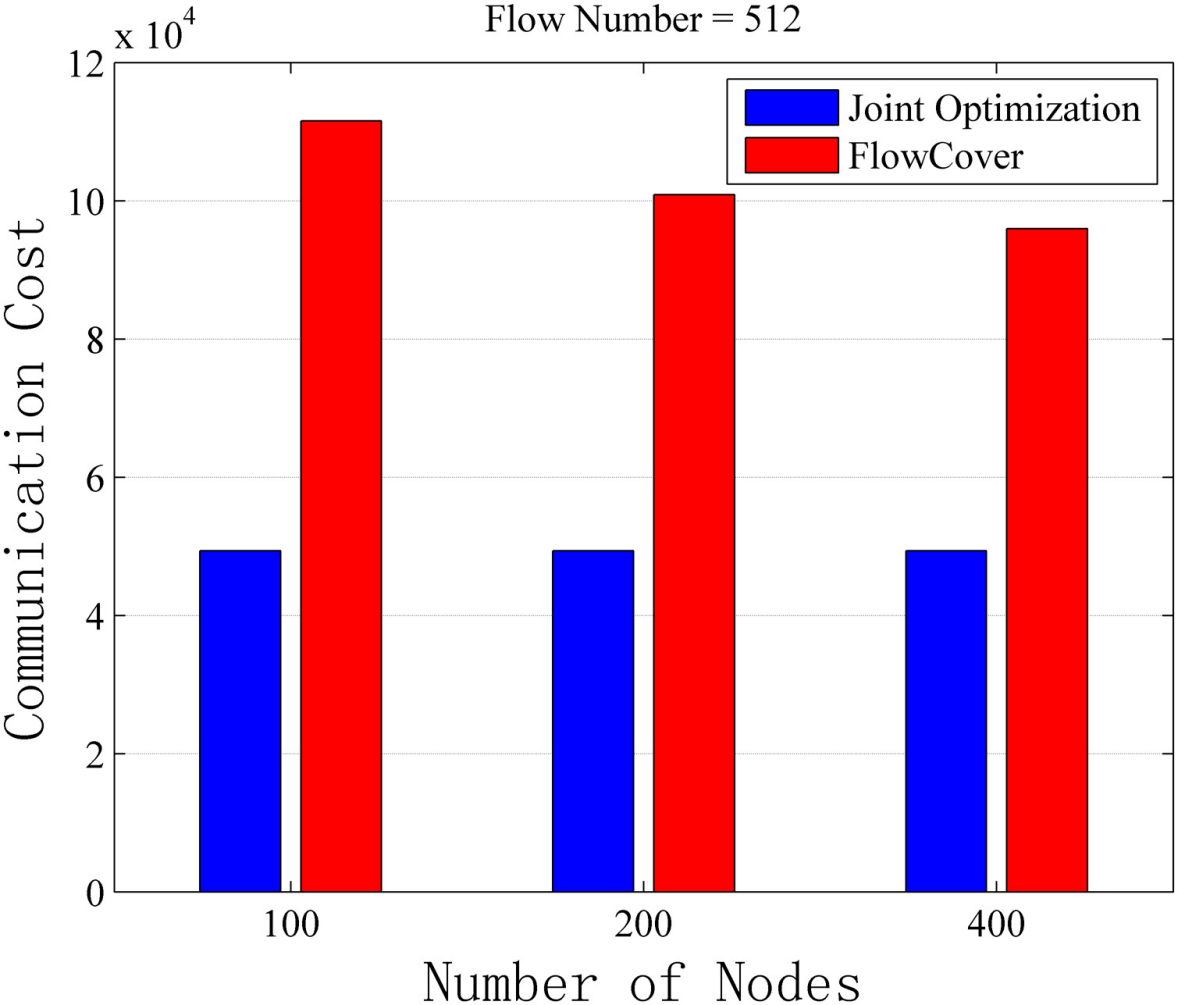
Communication Cost vs. Network Size on General Topologies with No Capacity Constraint. The simulation result with 512 flows and different number of nodes. The switch capacity is unlimited in this scenario.

**Fig 11:**
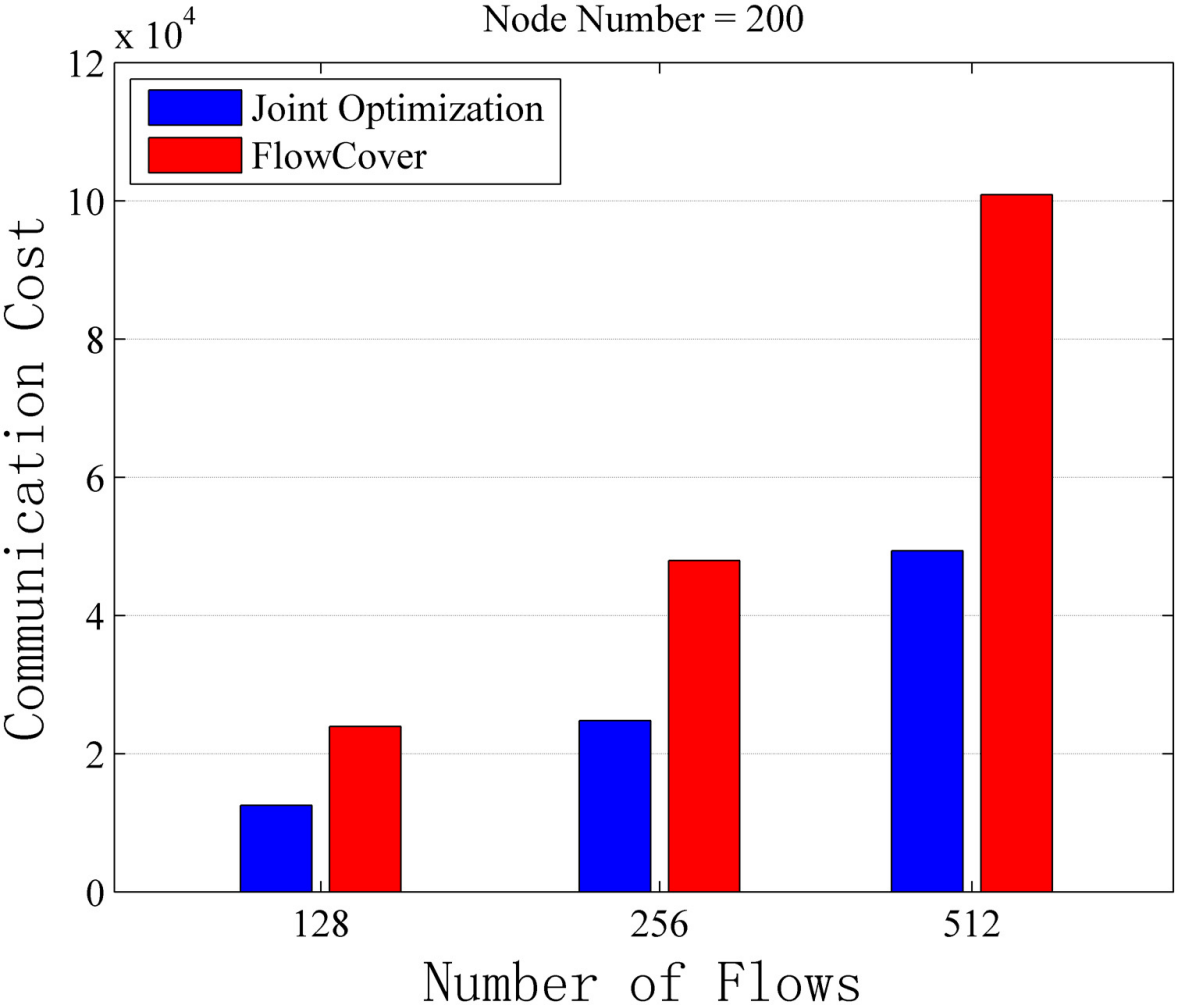
Communication Cost vs. Number of Flows on General Topologies with No Capacity Constraint. The simulation result with 200 nodes and different number of flows. The switch capacity is unlimited in this scenario.

At first, the number of flows is set to be 512 firstly, and the communication cost changes with network size is evaluated. Then, the number of nodes is set to 200, and the performance affected by the flow numbers is evaluated. Figs 10 and 11 shows the simulation results. From these figures, we make following main observations.

First of all, we observe that compared with FlowCover scheme, the joint optimization scheme proposed in our work can save 48.57%–55.76% communication cost with different numbers of nodes in the network, and save 47.86%–51.09% communication cost with different amounts of flow.

From Fig 10, we can see that the communication cost of FlowCover decreases when the number of nodes increases. The reason is that when the network size increases, there are less flows interleave with each other and the possibility of multiple monitoring is reduced. With fixed number of flows in the network, the communication cost may be the same in networks with different size if jointly optimization scheme is adopted. Accordingly, the communication cost keeps the same when the network size changes, and the only factor that affects the communication cost is the flow number in the network (as shown in Fig 11).

#### Performance with Limited Flow Table Capacity

**Fig 12:**
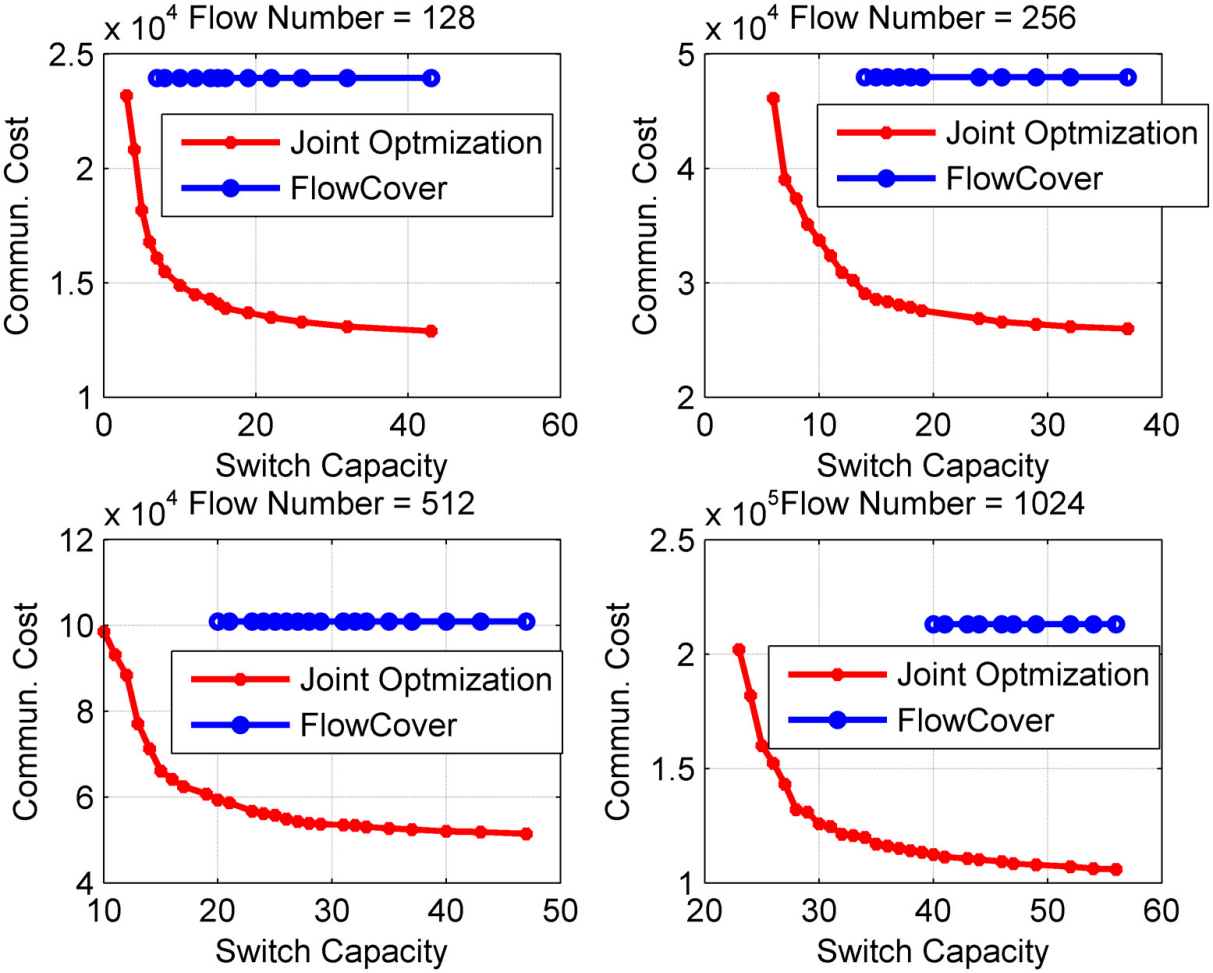
Performance on General Topologies vs. Switch Capacity Constraint. The simulation result with different switch capacities. The number of flows is set to a value in range of 128 to 1024. The number of nodes is 200.

To study the tradeoff between communication cost and the switch capacity, the number of switches is fixed to 200, and different numbers of flows are injected into the network. Fig 12 shows how the communication cost changes with different switch capacities. The detail results of the simulations can be found in S4 Appendix.

From this figure, we can see that the communication cost is lower with larger switch capacity. More importantly, the communication cost is very sensitive to the flow table capacity of switches when the capacity is relative small. For example, according to the first sub-figure of Fig 12, when the capacity changes from 1–20, the communication reduces about 40%. It means that the communication cost can be saved greatly with small switch capacity.

Furthermore, it is shown that by applying the switch capacity into the joint optimization scheme, the algorithm can not only get a balanced flow routing, but also reduce the communication cost for collecting flow information. This is due to the fact that FlowCover cannot always get the best routing in terms of the load balance, while SDN provides us the opportunity to optimize load balance and communication cost simultaneously by controlling the flow routing.

Comparing the performance on multi-rooted tree with that on general topology, we find that there is larger performance improvement on general topology. It is because that there are more routing choices on general topology, and hence there is larger optimization space to reduce the communication cost.

## Conclusion

This paper jointly optimizes flow routing and polling switch selection to reduce communication cost for collecting flow information in SDN. Besides formulating the joint optimization problem as an ILP model, an optimal algorithm for multi-rooted tree topology and an efficient heuristic for general topology is proposed. According to extensive simulations, it can be concluded that joint optimization scheme requires only about half of the cost to collect all the flow information in the networks compared with that of the latest switch-based scheme. It is a very promising result according to the high price and power consumption of hardware resources.

## Supporting Information

S1 Appendix The Solution of ILP Model and Algorithm 1 on Multi-Root Tree Topology. In S1 Appendix, we present the detail solutions of the ILP model proposed and that of Algorithm 1 in a PortLand topology with parameter *k* = 4.
(DOCX)

S2 Appendix The Detail of Simulation Results on Multi-Rooted Tree. S2 Appendix contains the detail results of simulations carried on multi-rooted tree topologies. In the results, different number of flows and different parameter *k* is applied. Moreover, in the limited flow table capacity scenario, the results with different flow table capacities are also presented.
(DOCX)

S3 Appendix The Distribution of Flows in the Solution of ILP Model and Algorithm 3. In S3 Appendix, we present the distribution of the flows used in the simulations.
(DOCX)

S4 Appendix The Detail of Simulation Results on General Topologies. S4 Appendix contains the detail results of simulations carried on general topologies. In the results, different number of flows and different number of nodes is applied. Moreover, in the limited flow table capacity scenario, the results with different flow table capacities are also presented.
(DOCX)

## References

[1] Moshref M, Yu M, Govindan R. Resource/Accuracy Tradeoffs in Software-Defined Measurement. HotSDN’13 Proceedings of the second ACM SIGCOMM workshop on hot topics in software defined networking. 2013; 1:73–78.

[2] Curtis AR, Mogul JC, Tourrilhes J, Yalagandula P, Sharma P, Banerjee S. DevoFlow: Scaling Flow Management for High-Performance Networks. ACM SIGCOMM Computer Communication Review. 2011; 41:254–265. 10.1145/2043164.2018466

[3] Jose L, Yu M, Rexford J. Online Measurement of Large Traffic Aggregates on Commodity Switches. Hot-ICE’11 Proceedings of the 11th USENIX conference on Hot topics in management of Internet, cloud, and enterprise networks and services. 2011; 1: 13–13.

[4] Yu M, Jose L, Miao R. Software Defined Traffic Measurement with OpenSketch. NSDI’13 Proceedings of the 10th USENIX conference on Networked Systems Design and Implementation. 2013; 1:29–42.

[5] Su Z, Wang T, Xia Y, Hamdi M. FlowCover: Low-cost Flow Monitoring Scheme in Software Defined Networks. IEEE Global Communications Conference (GLOBECOM). 2014; 1:1956–1961.

[6] Zhang Y. An Adaptive Flow Counting Method for Anomaly Detection in SDN. CoNEXT’13 Proceedings of the 9th ACM conference on Emerging networking experiments and technologies. 2013; 1:25–30.

[7] Yu M, Fabrikant A, Rexford J. BUFFALO: Bloom Filter Forwarding Architecture for Large Organizations. CoNEXT’09 Proceedings of the 5th ACM conference on Emerging networking experiments and technologies. 2009; 1:313–324.

[8] Yu M, Rexford J, Freedman MJ, Wang J. Scalable Flow-based Networking with DIFANE. ACM SIGCOMM Computer Communication Review. 2011; 41(4):351–362.

[9] Kanizo Y, Hay D, Keslassy I. Palette: Distributing Tables in Software-Defined Networks. INFOCOM, 2013 Proceedings IEEE. 2013; 1:515–549.

[10] Chowdhury SR, Bari MF, Ahmed R, Boutaba R. Payless: A Low Cost Network Monitoring Framework for Software Defined Networks. IEEE Network Operations and Management Symposium (NOMS). 2014; 1:1–9. 10.1109/NOMS.2014.6838227

[11] Kannan K, Banerjee S. Compact TCAM: Flow Entry Compaction in TCAM for Power Aware SDN Distributed Computing and Networking. Springer 2013.

[12] Estan C, Varghese G. New Directions in Traffic Measurement and Accounting. ACM SIGCOMM Computer Communication Review. 2002; 32(4):323–336 10.1145/964725.633056

[13] Sekar V, Reiter MK, Zhang H. Revisiting the Case for a Minimalist Approach for Network Flow Monitoring. Proceedings of the 10th ACM SIGCOMM Conference on Internet Measurement. ACM. 2010; 1:328–341.

[14] Claise, B. RFC3954 Cisco Systems NetFlow Services Export Version 9. 2004. Available: http://www.rfc-editor.org/rfc/rfc3954.txt.

[15] Wang M, Li B, Li Z. sFlow: Towards Resource-Efficient and Agile Service Federation in Service Overlay Networks. Proceedings of 24th International Conference on Distributed Computing Systems. 2004; 1:628–635.

[16] OpenFlow Consoritum. OpenFlow Switch Specification v1.0. 2009. Available: https://www.opennetworking.org/images/stories/downloads/sdn-resources/onf-specifications/openflow/openflow-spec-v1.0.0.pdf.

[17] Wu B, Ho PH, Yeung KL. Monitoring Trail: On Fast Link Failure Localization in All-Optical WDM Mesh Networks. Journal of Lightwave Technology. 2009; 27(18):4175–4185. 10.1109/JLT.2009.2022769

[18] Al-Fares M, Loukissas A, Vahdat A. A Scalable, Commodity Data Center Network Architecture. ACM SIGCOMM Computer Communication Review. 2008; 38(4):63–74. 10.1145/1402946.1402967

[19] IBM. CPLEX Optimizer. Available: http://www-01.ibm.com/software/commerce/optimization/cplex-optimizer/.

[20] White S, Smyth P. Algorithms for Estimating Relative Importance in Networks. Proceedings of the 9th ACM SIGKDD international conference on knowledge discovery and data mining. 2003; 1:266–275.

[21] Greenberg A, Hamilton JR, Jain N, Kandula S, Kim C, Lahiri P, et al VL2: A Scalable and Flexible Data Center Network. ACM SIGCOMM Computer Communication Review. 2009; 39: 51–62 10.1145/1594977.1592576

[22] Mills DL, Braun HW. The NSFNET Backbone Network. ACM SIGCOMM Computer Communication Review. 1987; 17: 191–196 10.1145/55483.55502

[23] Hagberg A, Schult DA, Swart PJ. NetworkX; 2013. Available: https://networkx.github.io/.

[24] Medina A, Taft N, Salamatian K, Bhattacharyya S, Diot C. Traffic Matrix Estimation: Existing Techniques and New Directions. ACM SIGCOMM Computer Communication Review. 2002; 32: 161–174 10.1145/964725.633041

