# Supplementary material for: A Scheme to Optimize Flow Routing and Polling Switch Selection of Software Defined Networks
Source: PLoS One. 2015 Dec 21;10(12):e0145437. doi: 10.1371/journal.pone.0145437 (PMC4686908; doi:10.1371/journal.pone.0145437)
Supplement: S1 Appendix — In S1 Appendix, we present the detail solutions of the ILP model proposed and that of Algorithm 1 in a PortLand topology with parameter k = 4. (DOCX) [file pone.0145437.s001.docx]

**S1 Appendix The Solutions of ILP Model and Algorithm 1 in Multi-Root Tree Topology**


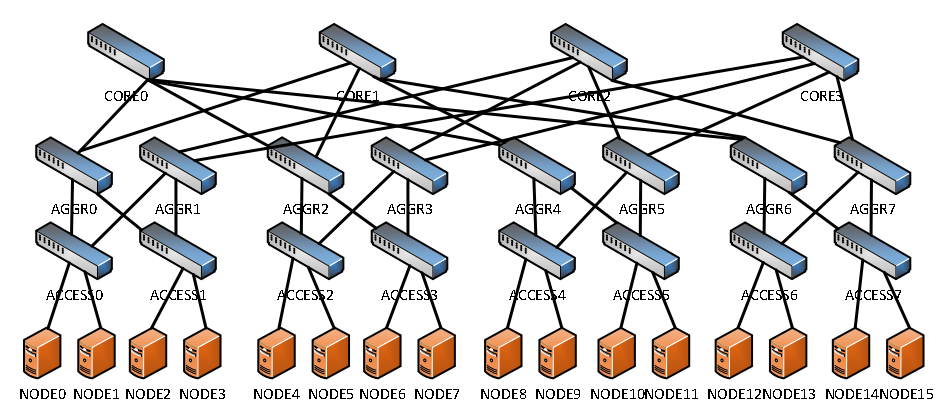


Fig.1. Multi-Root Tree Topology (k = 4)

1. **Inputs:**
2. Topology: The topology of multi-root tree with k = 4 is shown in Fig1.
3. Input flows: For a fair comparison, both ILP and Algorithm 1 used the same input flows, which are shown in Table 1.

**Table 1 Input Flows**

| No. | Source Node | Destination Node |
| --- | --- | --- |
| 1 | Node 15 | Node 6 |
| 2 | Node 12 | Node 1 |
| 3 | Node 3 | Node 5 |
| 4 | Node 0 | Node 11 |
| 5 | Node 4 | Node 2 |
| 6 | Node 14 | Node 7 |
| 7 | Node 8 | Node 10 |
| 8 | Node 12 | Node 9 |

To compare the solution of ILP model with Algorithm 1, we generate eight bidirectional flows on a Porland topology with the parameter k = 4, and ensure every host exactly has one flow. By applying both ILP model and Algorithm 1 on this scenario, we get the results shown in Table 1.

**Table 2 Performance Comparsion Between ILP Model and Algorithm 1**

| Algorithm | Switch capcity | Number of Polling Switches | | | Communication Cost |
| --- | --- | --- | --- | --- | --- |
|  |  | Core switch | Agg. Switch | Acc. Switch |  |
| ILP | 0 | 1 | 0 | 1 | 1168 |
| Alg. 1 | 0 | 1 | 1 | 0 | 1168 |
| ILP | 3 | 1 | 1 | 1 | 1368 |
| Alg. 1 | 3 | 2 | 1 | 0 | 1368 |

1. **Detail result of ILP solution**

**Table 3 Detail Result of ILP Solution with Unlimited Capacity**

| Flow No. | Route | Polling Switch |
| --- | --- | --- |
| 1 | NODE15-ACCESS7-AGGR6-CORE0-AGGR2-ACCESS3-NODE6 | CORE0 |
| 2 | NODE13-ACCESS6-AGGR6-CORE0-AGGR0-ACCESS0-NODE1 | CORE0 |
| 3 | NODE3-ACCESS1-AGGR0-CORE0-AGGR2-ACCESS2-NODE5 | CORE0 |
| 4 | NODE0-ACCESS0-AGGR1-CORE3-AGGR5-ACCESS5-NODE11 | ACCESS5 |
| 5 | NODE4-ACCESS2-AGGR2-CORE0-AGGR0-ACCESS1-NODE2 | CORE0 |
| 6 | NODE14-ACCESS7-AGGR6-CORE0-AGGR2-ACCESS3-NODE7 | CORE0 |
| 7 | NODE8-ACCESS4-AGGR4-ACCESS5-NODE10 | ACCESS5 |
| 8 | NODE12-ACCESS6-CORE0-AGGR4-ACCESS4-NODE9 | CORE0 |

**Table 4 Detail Result of ILP Solution with *capacity = 3***

| Flow No. | Route | Polling Switch |
| --- | --- | --- |
| 1 | NODE15-ACCESS7-AGGR7-CORE3-AGGR3-ACCESS3-NODE6 | AGGR7 |
| 2 | NODE13-ACCESS6-AGGR7-CORE3-AGGR1-ACCESS0-NODE1 | AGGR7 |
| 3 | NODE3-ACCESS1-AGGR0-CORE1-AGGR2-ACCESS2-NODE5 | CORE1 |
| 4 | NODE0-ACCESS0-AGGR1-CORE2-AGGR5-ACCESS5-NODE11 | ACCESS5 |
| 5 | NODE4-ACCESS2-AGGR2-CORE1-AGGR0-ACCESS1-NODE2 | CORE1 |
| 6 | NODE14-ACCESS7-AGGR7-CORE3-AGGR3-ACCESS3-NODE7 | AGGR7 |
| 7 | NODE8-ACCESS4-AGGR4-ACCESS5-NODE10 | ACCESS5 |
| 8 | NODE12-ACCESS6-CORE1-AGGR8-ACCESS4-NODE9 | CORE1 |

1. **Detail result of Algorithm 1**

**Table 5 Detail Result of Algorithm 1 with Unlimited Capcity**

| Flow No. | Route | Polling Switch |
| --- | --- | --- |
| 1 | NODE15-ACCESS7-AGGR6-CORE0-AGGR2-ACCESS3-NODE6 | CORE0 |
| 2 | NODE13-ACCESS6-AGGR6-CORE0-AGGR0-ACCESS0-NODE1 | CORE0 |
| 3 | NODE3-ACCESS1-AGGR0-CORE0-AGGR2-ACCESS2-NODE5 | CORE0 |
| 4 | NODE0-ACCESS0-AGGR0-CORE0-AGGR4-ACCESS5-NODE11 | CORE0 |
| 5 | NODE4-ACCESS2-AGGR2-CORE0-AGGR0-ACCESS1-NODE2 | CORE0 |
| 6 | NODE14-ACCESS7-AGGR6-CORE0-AGGR2-ACCESS3-NODE7 | CORE0 |
| 7 | NODE8-ACCESS4-AGGR5-ACCESS5-NODE10 | ACCESS5 |
| 8 | NODE12-ACCESS6-AGGR6-CORE0-AGGR4-ACCESS4-NODE9 | CORE0 |

**Table 6 Detail result of Algorithm 1 with *capacity = 3***

| Flow No. | Route | Polling Switch |
| --- | --- | --- |
| 1 | NODE15-ACCESS7-AGGR6-CORE0-AGGR2-ACCESS3-NODE6 | CORE0 |
| 2 | NODE13-ACCESS6-AGGR6-CORE0-AGGR0-ACCESS0-NODE1 | CORE0 |
| 3 | NODE3-ACCESS1-AGGR0-CORE0-AGGR2-ACCESS2-NODE5 | CORE0 |
| 4 | NODE0-ACCESS0-AGGR1-CORE2-AGGR5-ACCESS5-NODE11 | AGGR5 |
| 5 | NODE4-ACCESS2-AGGR2-CORE1-AGGR0-ACCESS1-NODE2 | CORE1 |
| 6 | NODE14-ACCESS7-AGGR6-CORE1-AGGR2-ACCESS3-NODE7 | CORE1 |
| 7 | NODE8-ACCESS4-AGGR5-ACCESS5-NODE10 | AGGR5 |
| 8 | NODE12-ACCESS6-AGGR7-CORE2-AGGR5-ACCESS4-NODE9 | AGGR5 |

The solution derived by ILP model and Algorithm 1 are different in terms of the flow routing and polling switch selection. However, according to the results from Table 3 – Table 6, we can see that the performance of Algorithm 1 is exactly the same as the optimal performance derived by ILP model in terms of the communication cost.
