# Supplementary material for: A Scheme to Optimize Flow Routing and Polling Switch Selection of Software Defined Networks
Source: PLoS One. 2015 Dec 21;10(12):e0145437. doi: 10.1371/journal.pone.0145437 (PMC4686908; doi:10.1371/journal.pone.0145437)
Supplement: S2 Appendix — contains the detail results of simulations carried on multi-rooted tree topologies. In the results, different number of flows and different parameter k is applied. Moreover, in the limited flow table capacity scenario, the results with different flow table capacities are also presented. (DOCX) [file pone.0145437.s002.docx]

**S2 Appendix The Detail Solutions of Algorithm 1**

**Table 1 Detail Result of Algorithm 1 and FlowCover with Unlimited Capcity**

| k | Number of Flows | FlowCover | Algorithm 1 | Optimization Efficiency |
| --- | --- | --- | --- | --- |
| 6 | 16 | 3072 | 2292 | 0.253906 |
|  | 32 | 6280 | 4208 | 0.329936 |
|  | 64 | 11624 | 8040 | 0.308328 |
|  | 128 | 25040 | 15000 | 0.400958 |
|  | 256 | 49824 | 28716 | 0.423651 |
|  | 512 | 101040 | 49344 | 0.511639 |
| 8 | 16 | 3420 | 2292 | 0.329825 |
|  | 32 | 5820 | 4396 | 0.244674 |
|  | 64 | 10864 | 8604 | 0.208027 |
|  | 128 | 21632 | 15328 | 0.29142 |
|  | 256 | 48868 | 30092 | 0.384219 |
|  | 512 | 99356 | 58516 | 0.411047 |
| 12 | 16 | 3608 | 1916 | 0.468958 |
|  | 32 | 6384 | 4208 | 0.340852 |
|  | 64 | 12988 | 7476 | 0.424392 |
|  | 128 | 22888 | 15516 | 0.32209 |
|  | 256 | 46796 | 29716 | 0.364988 |
|  | 512 | 90448 | 52056 | 0.424465 |
|  | 1024 | 207772 | 114352 | 0.449627 |
|  | 2048 | 447816 | 226824 | 0.493488 |
| 16 | 16 | 3984 | 2480 | 0.37751 |
|  | 32 | 6840 | 4020 | 0.412281 |
|  | 64 | 12984 | 7852 | 0.395256 |
|  | 128 | 22144 | 14088 | 0.363801 |
|  | 256 | 44784 | 27376 | 0.38871 |
|  | 512 | 88736 | 53152 | 0.40101 |
|  | 1024 | 182120 | 102904 | 0.434966 |
|  | 2048 | 386816 | 202808 | 0.475699 |
|  | 4096 | 791136 | 401264 | 0.4928 |

**Table 2 Detail Result of Algorithm 1 with Different Flow Table Capacity (*k = 16*)**

| Number of Flows | FlowCover | Flow Table Usage of Flow Cover | Algorithm 1 | Flow Table Capacity |
| --- | --- | --- | --- | --- |
| 128 | 22144 | 7 | 20888 | 3 |
|  |  |  | 18888 | 4 |
|  |  |  | 18088 | 5 |
|  |  |  | 17088 | 6 |
|  |  |  | 16888 | 7 |
|  |  |  | 16488 | 8 |
|  |  |  | 16088 | 9 |
|  |  |  | 15888 | 10 |
|  |  |  | 15688 | 11 |
|  |  |  | 15488 | 14 |
|  |  |  | 15088 | 17 |
|  |  |  | 14888 | 22 |
|  |  |  | 14688 | 28 |
|  |  |  | 14488 | 35 |
|  |  |  | 14288 | 56 |
|  |  |  | 14088 | 112 |
| 256 | 27376 | 10 | 40776 | 1 |
|  |  |  | 39976 | 2 |
|  |  |  | 39576 | 3 |
|  |  |  | 38976 | 4 |
|  |  |  | 35976 | 5 |
|  |  |  | 33976 | 6 |
|  |  |  | 33176 | 7 |
|  |  |  | 31976 | 8 |
|  |  |  | 31376 | 9 |
|  |  |  | 30776 | 10 |
|  |  |  | 30376 | 11 |
|  |  |  | 30176 | 12 |
|  |  |  | 29976 | 13 |
|  |  |  | 29576 | 14 |
|  |  |  | 29376 | 15 |
|  |  |  | 29176 | 17 |
|  |  |  | 28976 | 21 |
|  |  |  | 28776 | 24 |
|  |  |  | 28576 | 27 |
|  |  |  | 28376 | 33 |
|  |  |  | 28176 | 40 |
|  |  |  | 27976 | 53 |
|  |  |  |  |  |
| 512 | 53152 | 17 | 70952 | 1 |
|  |  |  | 67952 | 2 |
|  |  |  | 66152 | 3 |
|  |  |  | 65752 | 4 |
|  |  |  | 65552 | 5 |
|  |  |  | 64752 | 9 |
|  |  |  | 61552 | 10 |
|  |  |  | 60152 | 11 |
|  |  |  | 59152 | 12 |
|  |  |  | 58752 | 13 |
|  |  |  | 58152 | 14 |
|  |  |  | 57752 | 15 |
|  |  |  | 57552 | 16 |
|  |  |  | 57152 | 17 |
|  |  |  | 56952 | 18 |
|  |  |  | 56552 | 19 |
|  |  |  | 56352 | 20 |
|  |  |  | 56152 | 21 |
|  |  |  | 55952 | 22 |
|  |  |  | 55752 | 23 |
|  |  |  | 55552 | 24 |
|  |  |  | 55352 | 25 |
|  |  |  | 55152 | 27 |
|  |  |  | 54952 | 29 |
|  |  |  | 54752 | 30 |
|  |  |  | 54552 | 34 |
|  |  |  | 54352 | 35 |
|  |  |  | 54152 | 36 |
| 1024 | 102904 | 25 | 114920 | 12 |
|  |  |  | 114520 | 15 |
|  |  |  | 114120 | 16 |
|  |  |  | 111816 | 18 |
|  |  |  | 111008 | 19 |
|  |  |  | 109920 | 20 |
|  |  |  | 109712 | 21 |
|  |  |  | 109216 | 22 |
|  |  |  | 108920 | 23 |
|  |  |  | 108712 | 24 |
|  |  |  | 108320 | 25 |
|  |  |  | 108408 | 26 |
|  |  |  | 107912 | 27 |
|  |  |  | 107808 | 28 |
|  |  |  | 107608 | 29 |
|  |  |  | 107408 | 30 |
|  |  |  | 107304 | 31 |
|  |  |  | 107008 | 32 |
|  |  |  | 107104 | 34 |
|  |  |  | 106504 | 35 |
|  |  |  | 106312 | 36 |
|  |  |  | 106208 | 37 |
|  |  |  | 105912 | 38 |
