# Supplementary material for: A Scheme to Optimize Flow Routing and Polling Switch Selection of Software Defined Networks
Source: PLoS One. 2015 Dec 21;10(12):e0145437. doi: 10.1371/journal.pone.0145437 (PMC4686908; doi:10.1371/journal.pone.0145437)
Supplement: S3 Appendix — In S3 Appendix, we present the distribution of the flows used in the simulations. (DOCX) [file pone.0145437.s003.docx]

**S3 Appendix The Solutions of ILP Model and Algorithm 3 in NSFNET Topology**

1. **Inputs:**
2. Topology: The NSFNET topology.
3. Input flows: For a fair comparison, both ILP and Algorithm 3 used the same input flows, which are shown in Table 1-2

**Table 1 Input Flows (5 flows)**

| No. | Source Node | Destination Node |
| --- | --- | --- |
| 1 | NorthwestNet | Sesquinet |
| 2 | PSCNET | SURAnet |
| 3 | MIDnet | CNSF/NYSERNet |
| 4 | SDSCNET | JVNC |
| 5 | NCAR/USAN | BARRNet |

**Table 2Input Flows (8 flows)**

| No. | Source Node | Destination Node |
| --- | --- | --- |
| 1 | BARRNet | Merit |
| 2 | NorthwestNet | Sesquinet |
| 3 | NCSA/UIUC | PSCNET |
| 4 | SURAnet | NCAR/USAN |
| 5 | MIDnet | CNSF/NYSERNet |
| 6 | PSCNET | Westnet |
| 7 | Westnet | SDSCNET |
| 8 | NCAR/USAN | JVNC |

**Table 3 Input Flows (10 flows)**

| No. | Source Node | Destination Node |
| --- | --- | --- |
| 1 | Sesquinet | JVNC |
| 2 | CNSF/NYSERNet | BARRNet |
| 3 | BARRNet | SURAnet |
| 4 | NCAR/USAN | MIDnet |
| 5 | MIDnet | NorthwestNet |
| 6 | JVNC | PSCNET |
| 7 | PSCNET | Wesetnet |
| 8 | NCSA/UIUC | Merit |
| 9 | NorthwestNet | SDSCNET |
| 10 | SDSCNET | NCSA/UIUC |

1. **Detail result of ILP solution**

**Table 3 Detail Result of ILP Model with Unlimited Capcity (5 flows)**

| Flow No. | Route | Polling Switch |
| --- | --- | --- |
| 1 | NorthwestNet-SDSCNET-Sesquinet | Sesquinet |
| 2 | PSCNET-NCSA/UIUC-MIDnet-NCAR/USAN-Sesquinet-SDSCNET-NorthwesetNet-BARRNet-Westnet-Merit-CNSF/NYSERNet-SURAnet | Sesquinet |
| 3 | MIDnet-NCSA/UIUC-PSCNET-JVNC-Merit-Westnet-NCAR/USAN-Sesquinet-SURAnet-CNSF/NYSERNet | Sesquinet |
| 4 | SDSCNET-Sesquinet-NCAR/USAN-MIDnet-NCSA/UIUC-PSCNET-JVNC | Sesquinet |
| 5 | NCAR/USAN-Sesquinet-SDSCNET-BARRNet | Sesquinet |

**Table4 Detail Result of ILP Model with *capacity = 3* (5 flows)**

| Flow No. | Route | Polling Switch |
| --- | --- | --- |
| 1 | NorthwestNet-SDSCNET-Sesquinet-SURAnet-CNSF/NYSERNet | SURAnet |
| 2 | PSCNET-JVNC-Merit-CNSF/NYSERNet-SURAnet | SURAnet |
| 3 | SDSCNET-Sesquinet-NCAR/USAN-MIDnet-NCSA/UIUC-PSCNET-JVNC | MIDnet |
| 4 | NCAR/USAN-MIDnet-NCSA/UIUC-NorthwestNet-BARRNet t | MIDnet |
| 5 | MIDnet-NCAR/USAN-Westnet-Merit-CNSF/NYSERNet | MIDnet |

1. **Detail result of Algorithm 3**

**Table 5 Detail Result of Algorithm 3 with Unlimited Capcity (5 flows)**

| Flow No. | Route | Polling Switch |
| --- | --- | --- |
| 1 | NorthwesetNet-NCSA/UIUC-PSCNET-CNSF/NYSERNet-SURAnet-Sesquinet | PSCNET |
| 2 | PSCNET-CNSF/NYSERNet-SURAnet | PSCNET |
| 3 | MIDnet-NCSA/UIUC-PSCNET-CNSF/NYSERNet | PSCNET |
| 4 | SDSCNET-NorthwesteNet-NCSA/UIUC-PSCNET-JVNC | PSCNET |
| 5 | NCAR/USAN-MIDnet-NCSA/UIUC-PSCNET-NCS/UIUC-NorthwestNet-BARRNet | PSCNET |

**Table 6 Detail Result of Algorithm 3 with *capacity = 3* (5 flows)**

| Flow No. | Route | Polling Switch |
| --- | --- | --- |
| 1 | NorthwestNet-BARRNet-Westnet-NCAR/USAN-Sesquinet | Westnet |
| 2 | PSCNET-CNSF/NYSERNet-SURAnet | PSCNET |
| 3 | MIDnet-NCSA/UIUC-PSCNET-CNSF/NYSERNet | PSCNET |
| 4 | SDSCNET-BARRNet-Westnet-Merit-JVNC | Westnet |
| 5 | NCAR/USAN-Westnet-BARRNet | Westnet |
