# Supplementary material for: A Scheme to Optimize Flow Routing and Polling Switch Selection of Software Defined Networks
Source: PLoS One. 2015 Dec 21;10(12):e0145437. doi: 10.1371/journal.pone.0145437 (PMC4686908; doi:10.1371/journal.pone.0145437)
Supplement: S4 Appendix — contains the detail results of simulations carried on general topologies. In the results, different number of flows and different number of nodes is applied. Moreover, in the limited flow table capacity scenario, the results with different flow table capacities are also presented. (DOCX) [file pone.0145437.s004.docx]

**S4 Appendix The Detail Solutions of Algorithm 3**

**Table 1 Detail Result of Algorithm 3 and FlowCover with Unlimited Capcity**

| Number of Nodes | Number of Flows | FlowCover | Algorithm 3 | Optimization Efficiency |
| --- | --- | --- | --- | --- |
| 100 | 16 | 3904 | 1916 | 0.509221 |
|  | 32 | 6896 | 3644 | 0.471578 |
|  | 64 | 12988 | 7100 | 0.453342 |
|  | 128 | 27048 | 14012 | 0.481958 |
|  | 256 | 55364 | 27836 | 0.497218 |
|  | 512 | 111552 | 49352 | 0.557587 |
| 200 | 16 | 3904 | 1916 | 0.509221 |
|  | 32 | 6840 | 3644 | 0.467251 |
|  | 64 | 13820 | 7100 | 0.486252 |
|  | 128 | 23952 | 14012 | 0.414996 |
|  | 256 | 47960 | 27836 | 0.419600 |
|  | 512 | 100896 | 49352 | 0.510863 |
|  | 1024 | 213112 | 98504 | 0.537783 |
| 400 | 16 | 3796 | 1916 | 0.495258 |
|  | 32 | 7028 | 3644 | 0.481503 |
|  | 64 | 12956 | 7100 | 0.451991 |
|  | 128 | 25920 | 14012 | 0.459414 |
|  | 256 | 49036 | 27836 | 0.432335 |
|  | 512 | 95960 | 49352 | 0.485702 |
|  | 1024 | 197808 | 98504 | 0.502022 |
|  | 2048 | 467408 | 221372 | 0.526384 |

**Table 2 Detail Result of Algorithm 3 with Different Flow Table Capacity (200 nodes)**

| Number of Flows | FlowCover | Flow Table Usage of Flow Cover | Algorithm 3 | Flow Table Capacity |
| --- | --- | --- | --- | --- |
| 128 | 23952 | 7 | 23176 | 3 |
|  |  |  | 20824 | 4 |
|  |  |  | 18168 | 5 |
|  |  |  | 16784 | 6 |
|  |  |  | 16088 | 7 |
|  |  |  | 15488 | 8 |
|  |  |  | 14888 | 10 |
|  |  |  | 14488 | 12 |
|  |  |  | 14288 | 14 |
|  |  |  | 14088 | 15 |
|  |  |  | 13888 | 16 |
|  |  |  | 13688 | 19 |
|  |  |  | 13488 | 22 |
|  |  |  | 13288 | 26 |
|  |  |  | 13088 | 32 |
|  |  |  | 12888 | 43 |
| 256 | 47960 | 14 | 46128 | 6 |
|  |  |  | 39032 | 7 |
|  |  |  | 37360 | 8 |
|  |  |  | 35112 | 9 |
|  |  |  | 33736 | 10 |
|  |  |  | 32368 | 11 |
|  |  |  | 30904 | 12 |
|  |  |  | 30216 | 13 |
|  |  |  | 29048 | 14 |
|  |  |  | 28560 | 15 |
|  |  |  | 28360 | 16 |
|  |  |  | 28064 | 17 |
|  |  |  | 27864 | 18 |
|  |  |  | 27568 | 19 |
|  |  |  | 26872 | 24 |
|  |  |  | 26576 | 26 |
|  |  |  | 26376 | 29 |
|  |  |  | 26176 | 32 |
|  |  |  | 25976 | 37 |
| 512 | 100896 | 20 | 98568 | 10 |
|  |  |  | 93152 | 11 |
|  |  |  | 88424 | 12 |
|  |  |  | 77112 | 13 |
|  |  |  | 71304 | 14 |
|  |  |  | 66080 | 15 |
|  |  |  | 64136 | 16 |
|  |  |  | 62480 | 17 |
|  |  |  | 60720 | 19 |
|  |  |  | 59360 | 20 |
|  |  |  | 58680 | 21 |
|  |  |  | 56736 | 23 |
|  |  |  | 56152 | 24 |
|  |  |  | 55760 | 25 |
|  |  |  | 54888 | 26 |
|  |  |  | 54304 | 27 |
|  |  |  | 53912 | 28 |
|  |  |  | 53712 | 29 |
|  |  |  | 53512 | 31 |
|  |  |  | 53416 | 32 |
|  |  |  | 53024 | 33 |
|  |  |  | 52728 | 35 |
|  |  |  | 52432 | 37 |
|  |  |  | 52040 | 40 |
|  |  |  | 51840 | 43 |
|  |  |  | 51448 | 47 |
| 1024 | 213122 | 40 | 202024 | 23 |
|  |  |  | 181896 | 24 |
|  |  |  | 159928 | 25 |
|  |  |  | 152312 | 26 |
|  |  |  | 143152 | 27 |
|  |  |  | 132072 | 28 |
|  |  |  | 130904 | 29 |
|  |  |  | 125792 | 30 |
|  |  |  | 124632 | 31 |
|  |  |  | 121256 | 32 |
|  |  |  | 120656 | 33 |
|  |  |  | 119952 | 34 |
|  |  |  | 116904 | 35 |
|  |  |  | 116128 | 36 |
|  |  |  | 115160 | 37 |
|  |  |  | 114096 | 38 |
|  |  |  | 113416 | 39 |
|  |  |  | 112448 | 40 |
|  |  |  | 111288 | 41 |
|  |  |  | 110704 | 43 |
|  |  |  | 110216 | 44 |
|  |  |  | 109344 | 46 |
|  |  |  | 108376 | 47 |
|  |  |  | 107888 | 49 |
|  |  |  | 107112 | 52 |
|  |  |  | 106240 | 54 |
|  |  |  | 105944 | 56 |
